# Supplementary material for: Prevention of dementia using mobile phone applications (PRODEMOS): protocol for an international randomised controlled trial
Source: BMJ Open. 2021 Jun 9;11(6):e049762. doi: 10.1136/bmjopen-2021-049762 (PMC8191602; doi:10.1136/bmjopen-2021-049762)
Supplement: Supplementary data [file bmjopen-2021-049762supp001.pdf]

Prevention of Dementia using Mobile phone Applications (PRODEMOS): A randomised implementation trial

PARTICIPANT CONSENT FORM

Identification number: ________________________

Prevention Of Dementia Using Mobile Phone Applications (PRODEMOS)

SAMPLE

	Please initial
I confirm that I have read and understood the information sheet (Participant Information Sheet Version 1.1 dated 19/10/2020) for the study: **Prevention of Dementia using Mobile Phone applications**	☐
I have had enough time to ask questions. My questions were answered clearly. I have had enough time to consider participating in the study.	☐
I am aware that participation is voluntary. I am aware that I can withdraw at any stage. I do not need to give a reason.	☐
I understand that my medical care and legal rights will not be affected in any way.	☐
I understand why my data is being collected, and I give permission to use these data for the study purposes as mentioned in the information letter.	☐
I agree to giving a blood sample (fingerprick) for cholesterol testing.	☐
I am aware that I am able to pause the intervention, should my life circumstances change significantly.	☐
I am happy for my GP to know that I am participating in the PRODEMOS study, and for any examination results to be shared with my GP.	☐
I am happy that my data will be stored securely, and I give permission for my data to be stored and managed as mentioned in the participant information sheet.	☐
I am aware that I can ask for my data to be destroyed, prior to 31st December 2022.	☐
I do / do not* give permission to contact me about future research projects.	☐
I am willing to participate in this study.	☐

This project has received funding from the European Union's Horizon 2020 research and innovation programme under grant agreement No 779238

I would like to receive a copy of the results of this study.<br>(e-mail or postal address):	☐

Name of participant:

Signature of participant:

Date: __ / __ / ____

* Please delete as appropriate

Hereby I declare that I have fully informed the participant about the current research, encouraged the participant to ask questions, and provided adequate time to answer them. In the case of new information that could influence the permission of the participant, I will inform the participant in a timely fashion. I further ensure that 2 copies of the consent form have been completed: 1 copy to be left with the participant, and 1 copy to be filed with the researcher.

Name of researcher:

Signature: Date: __ / __ / ____

2 consent forms to be signed by both parties: 1 copy for the participant; 1 copy for the researcher site file.

SAMPLE

This project has received funding from the European Union's Horizon 2020 research and innovation programme under grant agreement No 779238

Prevention of Dementia using Mobile phone Applications

Medical Records Consent

Identification number: |__|-|__|__|__|-|__|__|__|__|

Please Initial

I confirm that I have read and understood the medical records information sheet version 05 dated 04/08/2020 for the PRODEMOS Project and have had the opportunity to ask questions.	☐
I give my permission to PRODEMOS to share my screening, baseline and final outcome visit results with my GP for inclusion in my NHS health record.	☐
I agree to the research staff on the PRODEMOS project accessing my GP medical records.	☐
I agree to PRODEMOS informing my GP of any abnormal test results taken at the screening, baseline, or final visits with the PRODEMOS study.	☐
I understand that my participation will not affect my medical care or legal rights in any way.	☐

SAMPLE

Name of Participant:

Signature of Participant

Date: ___ / ___ / _____

Hereby I declare that I have fully informed the participant about the current research, encouraged the participant to ask questions, and provided adequate time to answer them. In the case of new information that could influence the permission of the participant, I will inform the participant in a timely fashion. I further ensure that 2 copies of the consent form have been completed: 1 copy to be left with the participant, and 1 copy to be filed with the researcher.

This project has received funding from the European Union's Horizon 2020 research and innovation programme under grant agreement No 779238

Name of person receiving consent:<br>Signature:<br>Job Title: Date: ___ / ___ / ______

2 consent forms to be signed by both parties: 1 copy for the participant; 1 copy for the researcher site file

SAMPLE

This project has received funding from the European Union's Horizon 2020 research and innovation programme under grant agreement No 779238
