# Supplementary material for: Prevention of dementia using mobile phone applications (PRODEMOS): protocol for an international randomised controlled trial
Source: BMJ Open. 2021 Jun 9;11(6):e049762. doi: 10.1136/bmjopen-2021-049762 (PMC8191602; doi:10.1136/bmjopen-2021-049762)
Supplement: Author's manuscript [file bmjopen-2021-049762.draft_revisions.pdf]

BMJ Open

BMJ Open is committed to open peer review. As part of this commitment we make the peer review history of every article we publish publicly available.

When an article is published we post the peer reviewers’ comments and the authors’ responses online. We also post the versions of the paper that were used during peer review. These are the versions that the peer review comments apply to.

The versions of the paper that follow are the versions that were submitted during the peer review process. They are not the versions of record or the final published versions. They should not be cited or distributed as the published version of this manuscript.

BMJ Open is an open access journal and the full, final, typeset and author-corrected version of record of the manuscript is available on our site with no access controls, subscription charges or pay-per-view fees (http://bmjopen.bmj.com).

If you have any questions on BMJ Open’s open peer review process please email info.bmjopen@bmj.com

BMJ Open

Prevention of Dementia using Mobile Phone Applications (PRODEMOS): an international randomised controlled trial

Journal:	*BMJ Open*
Manuscript ID	bmjopen-2021-049762
Article Type:	Protocol
Date Submitted by the Author:	02-Feb-2021
Complete List of Authors:	Eggink, Esmé; Amsterdam UMC Locatie AMC, Department of General Practice<br>Hafdi, Melanie; Amsterdam UMC Locatie AMC, Department of Neurology<br>Hoevenaar-Blom, Marieke ; Amsterdam UMC Locatie AMC, Department of Neurology<br>Song, Manshu; Capital Medical University School of Public Health, Beijing Key Laboratory of Clinical Epidemiology; Edith Cowan University School of Medical and Health Sciences<br>Andrieu, Sandrine; INSERM-University of Toulouse UMR1027; Toulouse University Hospital, Department of Epidemiology and Public Health<br>Barnes, Linda E; University of Cambridge Institute of Public Health, Department of Public Health and Primary Care<br>Birck, Cindy; Alzheimer Europe<br>Brooks, Rachael L; University of Cambridge Institute of Public Health, Department of Public Health and Primary Care<br>Coley, Nicola; INSERM-University of Toulouse UMR1027; Toulouse University Hospital, Department of Epidemiology and Public Health<br>Ford, Elizabeth; Brighton and Sussex Medical School, Department of Primary Care and Public Health<br>Georges, Jean; Alzheimer Europe<br>van der Groep, Abraham; Vital Health Software<br>van Gool, Willem A; Amsterdam UMC Locatie AMC, Department of Public and Occupational Health<br>Handels, Ron; School for Mental Health and Neuroscience, Maastricht University Medical Centre+, Alzheimer Centre Limburg; Karolinska Institutet, Division of Neurogeriatrics, Department of Neurobiology, Care Science and Society<br>HOU, Haifeng; Edith Cowan University School of Medical and Health Sciences; Shandong First Medical University & Shandong Academy of Medical Science, School of Public Health<br>Li, Dong; Shandong First Medical University & Shandong Academy of Medical Science, School of Public Health<br>Liu, Hongmei; Beijing Neurosurgical Institute<br>Lyu, Jihui; Beijing Geriatric Hospital, Center for Cognitive Disorders<br>van Marwijk, Harm; Brighton and Sussex Medical School, Department of Primary Care and Public Health<br>van der Meijden, Mark; Vital Health Software<br>Niu, Yixuan; The Second Medical Centre & National Clinical Research Centre for Geriatric Diseases, Chinese PLA General Hospital, Department of Geriatrics<br>Sadhwani, Shanu; Brighton and Sussex Medical School, Department of

Primary Care and Public Health
Wang, Wenzhi; Beijing Neurosurgical Institute
Wang, Youxin; Capital Medical University School of Public Health, Beijing Key Laboratory of Clinical Epidemiology; Edith Cowan University School of Medical and Health Sciences
Wimo, Anders; Karolinska Institutet, Division of Neurogeriatrics, Department of Neurobiology, Care Sciences and Society
Ye, Xiaoyan; Fuzhou Comvee Network & Technology Co., Ltd
Yu, Yueyi; Xuanwu Hospital Capital Capital Medical University, Department of Neurology
Zeng, Qiang; The Second Medical Centre & National Clinical Research Centre for Geriatric Diseases, Chinese PLA General Hospital, Health Management Institute
Zhang, Wei; Beijing Tiantan Hospital, Capital Medical University, Centre for Cognitive Neurology, Department of Neurology
Wang, Wei; Capital Medical University School of Public Health, Beijing Key Laboratory of Clinical Epidemiology; Edith Cowan University School of Medical and Health Sciences
Brayne, Carol; University of Cambridge Institute of Public Health, Department of Public Health and Primary Care
Moll van Charante, Eric P; Amsterdam UMC Locatie AMC, Department of General Practice; Amsterdam UMC Locatie AMC, Department of Public Health
Richard, Edo; Amsterdam UMC Locatie AMC, Department of Public and Occupational Health; Radboud University Donders Institute for Brain Cognition and Behaviour, Department of Neurology

Keywords: Dementia < NEUROLOGY, Adult neurology < NEUROLOGY, PUBLIC HEALTH

SCHOLARONE™
Manuscripts

I, the Submitting Author has the right to grant and does grant on behalf of all authors of the Work (as defined in the below author licence), an exclusive licence and/or a non-exclusive licence for contributions from authors who are: i) UK Crown employees; ii) where BMJ has agreed a CC-BY licence shall apply, and/or iii) in accordance with the terms applicable for US Federal Government officers or employees acting as part of their official duties; on a worldwide, perpetual, irrevocable, royalty-free basis to BMJ Publishing Group Ltd ("BMJ") its licensees and where the relevant Journal is co-owned by BMJ to the co-owners of the Journal, to publish the Work in this journal and any other BMJ products and to exploit all rights, as set out in our licence.

The Submitting Author accepts and understands that any supply made under these terms is made by BMJ to the Submitting Author unless you are acting as an employee on behalf of your employer or a postgraduate student of an affiliated institution which is paying any applicable article publishing charge ("APC") for Open Access articles. Where the Submitting Author wishes to make the Work available on an Open Access basis (and intends to pay the relevant APC), the terms of reuse of such Open Access shall be governed by a Creative Commons licence – details of these licences and which Creative Commons licence will apply to this Work are set out in our licence referred to above.

Other than as permitted in any relevant BMJ Author's Self Archiving Policies, I confirm this Work has not been accepted for publication elsewhere, is not being considered for publication elsewhere and does not duplicate material already published. I confirm all authors consent to publication of this Work and authorise the granting of this licence.

For peer review only

Protocol article

Prevention of Dementia using Mobile Phone Applications (PRODEMOS): an international randomised controlled trial

Esmé Eggink[1], Melanie Hafdi[2], Marieke P Hoevenaar-Blom[2], Manshu Song[3,4], Sandrine Andrieu[5,6], Linda E Barnes[7], Cindy Birck[8], Rachael L Brooks[7], Nicola Coley[5,6], Elizabeth Ford[9], Jean Georges[8], Abraham van der Groep[10], Willem A van Gool[11], Ron LH Handels[12,13], Haifeng Hou[4,14], Dong Li[14], Hongmei Liu[15], Jihui Lyu[16], Harm WJ van Marwijk[9], Mark van der Meijden[10], Yixuan Niu[17], Shanu Sadhwani[9], Wenzhi Wang[15], Youxin Wang[3,4], Anders Wimo[13], Xiaoyan Ye[18], Yueyi Yu[19], Qiang Zeng[20], Wei Zhang[21], Wei Wang[3,4,14]*, Carol Brayne[7]*, Eric P Moll van Charante[1,22]*, Edo Richard[11,23]*#

*Shared last authors

#Corresponding author:
Dr. Edo Richard
Meibergdreef 9, 1105 AZ Amsterdam
e.richard@amsterdamumc.nl
0205669111

Author affiliations

[1] Department of General Practice, Amsterdam University Medical Centre, University of Amsterdam, Amsterdam, The Netherlands
[2]Department of Neurology, Amsterdam University Medical Centre, University of Amsterdam, Amsterdam, The Netherlands
[3]Beijing Key Laboratory of Clinical Epidemiology, School of Public Health, Capital Medical University, Beijing, China
[4]School of Medical and Health Sciences, Edith Cowan University, Perth, Australia
[5]INSERM/University of Toulouse UMR1027, Toulouse, France
[6]Department of Epidemiology and Public Health, Toulouse University Hospital, Toulouse, France
[7]Department of Public Health and Primary Care, Cambridge Institute of Public Health, University of Cambridge, Cambridge, United Kingdom
[8]Alzheimer Europe, Luxemburg City, Luxemburg
[9]Department of Primary Care and Public Health, Brighton and Sussex Medical School, Brighton, United Kingdom
[10]Vital Health Software, Ede, Netherlands
[11]Department of Public and Occupational Health, Amsterdam University Medical Centre, University of Amsterdam, Amsterdam, The Netherlands
[12]Alzheimer Centre Limburg, School for Mental Health and Neuroscience, Maastricht University Medical Centre+, Maastricht, the Netherlands
[13]Division of Neurogeriatrics, Department of Neurobiology, Care Sciences and Society, Karolinska Institutet, Stockholm, Sweden
[14]School of Public Health, Shandong First Medical University & Shandong Academy of Medical Science, Tai'an, Shandong, China
[15]Beijing Neurosurgical Institute, Beijing, China
[16]Centre for Cognitive Disorders, Beijing Geriatric Hospital, Beijing, China
[17] Department of Geriatrics, The Second Medical Centre & National Clinical Research Centre for Geriatric Diseases, Chinese PLA General Hospital, Beijing, China
[18]Fuzhou Comvee Network & Technology Co., Ltd, Fuzhou, China
[19]Department of Neurology, Xuanwu Hospital, Capital Medical University, Beijing, China
[20]Health Management Institute, The Second Medical Centre & National Clinical Research Centre for Geriatric Diseases, Chinese PLA General Hospital, Beijing, China
[21]Centre for Cognitive Neurology, Department of Neurology, Beijing Tiantan Hospital, Capital Medical University, Beijing, China
[22]Department of Public Health, Amsterdam University Medical Centre, University of Amsterdam, Amsterdam, The Netherlands
[23]Department of Neurology, Donders Institute for Brain, Behaviour and Cognition, Radboud University Medical Centre, Nijmegen, The Netherlands

Key words: Dementia; prevention; lifestyle intervention; mobile health
Word count: 4000 (excluding title page, abstract and strength and limitations section)

For peer review only

Abstract

Introduction Profiles of high risk for future dementia are well understood and are likely to concern mostly those in low- and middle-income countries, and people at greater disadvantage in high-income countries. Approximately 30-40% of all dementia cases have been estimated to be attributed to modifiable risk factors, including hypertension, smoking, and sedentary lifestyle. Tailored interventions targeting these risk factors have the potential to prevent or delay the onset of dementia. Mobile Health (mHealth) improves accessibility of such prevention strategies in hard-to-reach populations, while at the same time tailoring such approaches. In the current study we will investigate the effectiveness and implementation of a coach-supported mHealth intervention, targeting dementia risk factors, to reduce dementia risk.

Methods and analysis The PRODEMOS RCT will follow an effectiveness-implementation hybrid design, taking place in the United Kingdom (UK) and China. People are eligible if they are 55-75 years, of low socioeconomic status (UK) or from the general population (China), have ≥2 dementia risk factors, and own a smartphone. Overall, 2400 participants will be randomised to either a coach-supported, interactive mHealth platform, facilitating self-management of dementia risk factors, or to a static control platform. The intervention and follow-up period will be 18 months. The primary effectiveness outcome is change in the previously validated CAIDE dementia risk score. Main secondary outcomes include improvement of individual risk factors and cost-effectiveness. Implementation outcomes include acceptability, adoption, feasibility, and sustainability of the intervention.

Ethics and dissemination The PRODEMOS trial is sponsored in the UK by the University of Cambridge and is granted ethical approval by the London – Brighton&Sussex Research Ethics Committee (REC Reference: 20/LO/01440). In China the trial is approved by the Medical Ethics Committees of Capital Medical University (CMU), Beijing Tiantan Hospital, Beijing Geriatric Hospital, Chinese People's Liberation Army General Hospital, Taishan Medical University, and Xuanwu Hospital.

Trial registration number: ISRCTN15986016

Strengths and limitations of this study

- The coach-supported mHealth intervention builds on the previously developed HATICE electronic health (eHealth) platform, which was shown to improve cardiovascular risk of elderly in the Netherlands, France, and Finland.

- The main strengths of the present study are: testing the approach in populations with low socioeconomic status or different cultural settings and measuring whether sustained involvement of end-users can be achieved, facilitating cultural adaptation of the application.

- Limitations of this study include the impossibility to completely blinding the participants, potentially leading to contamination, and the challenge to sufficiently engage the hard-to-reach target population.

For peer review only

Introduction

With global ageing the prevalence of dementia is expected to increase to over 130 million in 2050, especially in low- and middle-income countries (LMIC) and in people from low socioeconomic status (SES) background in high-income countries (HIC)[1 2]. Strategies need to be developed that aim to reduce the risk of dementia – many of which will be at community and population level, but those that are individually based must be effective, affordable, and easily implementable across various healthcare settings.

Up to 40% of dementia cases are estimated to be attributable to potentially modifiable risk factors[3], of which 10-20% are cardiovascular risk factors including hypertension, midlife obesity, dyslipidaemia, diabetes, smoking, and physical inactivity[4-6]. To date, intervention studies aiming to reduce dementia risk by targeting one or more of these risk factors have shown inconsistent results[7 8]. Results from randomised controlled trial (RCTs) on blood pressure lowering treatment have suggested a beneficial effect on dementia risk, although not consistently and convincingly[9-11]. Since the presence of multiple risk factors may pose an additive or even synergistic effect on dementia risk[12 13], targeting several risk factors simultaneously may be more effective. The only study to date designed to address this question using dementia as primary outcome did not show a statistically significant effect after 6-8 years of intervention, although subgroup analysis suggested benefit for those with untreated hypertension at baseline[14].

A considerable challenge when designing a dementia prevention trial is the time lag between the optimal timing of the intervention and the onset of dementia. Using incident dementia as primary outcome requires large sample sizes and/or long follow-up periods to reach statistical power[15 16]. Dementia risk scores could be used as a proxy, especially in trials with follow-up periods up to several years. Another challenge, possibly explaining the neutral results of intervention studies so far, is the small window for risk factor improvement given a background of high-quality cardiovascular risk management in HIC where these studies were performed[17]. This lends further support for targeting people in LMIC and low SES populations in HIC.

Digital health interventions have the potential to improve cardiovascular risk factors in middle-age and beyond, especially when offered with human coaching (blended care)[18]. In the Healthy Ageing Through Internet Counselling in the Elderly (HATICE) trial, we recently demonstrated that a coach-supported internet intervention to optimise self-management of cardiovascular risk factors can reduce older adult's cardiovascular risk over a static control platform, both in high and low socioeconomic participant subgroups[19]. Currently, digital health interventions are increasingly offered through smartphones. Approximately 50% of the LMIC population and 67% of people with the lowest SES in UK owned a smartphone in 2018[20-22], and rates are increasing. This renders mobile health (mHealth) a

For peer review only

promising method for health delivery in underserved populations, including the improvement of cardiovascular risk factors[23 24].

We have developed a coach-supported mHealth intervention to reduce dementia risk by addressing common cardiovascular risk factors via lifestyle changes, building on the existing HATICE internet platform. Our aim is to assess the effectiveness and implementation of this smartphone intervention on dementia risk in older people at increased risk of dementia from a low SES population in the UK and from the general population in Beijing, China.

For peer review only

Methods

Study design

PRODEMOS is a multinational, prospective, open-label blinded endpoint (PROBE) trial with 18 months intervention and follow-up. The study follows a hybrid effectiveness-implementation design, taking a dual focus on assessing effectiveness and implementation outcomes[25 26]. The Amsterdam University Medical Centre (Amsterdam UMC) is the coordinating centre.

Study population and recruitment

The study population will consist of community-dwelling older adults aged 55-75 years, of low SES in the UK and of any SES in China, who have ≥2 dementia risk factors and own a smartphone. Low SES in the UK is operationalised as living in a postal code area ranked as equal to or less than the lowest third decile of the index of multiple deprivation[27]. Eligibility criteria are similar for both countries, except for criteria for obesity, based on differences in national prevention guidelines[28] (table 1).

Recruitment will take place in the Eastern Clinical Research Network (National Institutes of Health Research) region of the UK and in the Beijing and Tai'an cities, China. In the UK, recruitment will be undertaken in approximately 10-15 general practices (GPs). A random computer selection of participants living in the designated postal code areas meeting the age criterion and having ≥1 known dementia risk factor according to the GP registry will be approached through an information letter, inviting them to contact the local study centre. In China, participants will be recruited from seven hospitals through advertisements on hospital websites, targeted recruitment via local social media (WeChat), or direct approach by nurses and physicians. Recruitment is expected to start in early 2021.

Intervention and control condition

Central to our study is the PRODEMOS platform, which interconnects the assessor portal, participant app, and the coach portal (Figure 1). The assessor portal facilitates blinded collection of baseline and outcome assessments for all participants. The intervention and control condition are both delivered through a smartphone app, which, in the case of participants randomised to the intervention condition, allows communication with the coach portal. Data from the assessor portal, participant app, and coach portal can be extracted through a researcher portal and stored in a central database. The PRODEMOS platform was built in close collaboration between software developers and researchers from Amsterdam UMC, University of Cambridge, Brighton and Sussex Medical School, Capital Medical University in Beijing, health coaches, and representatives of the target population from both countries. The internet platform previously used in the HATICE trial served as the basis for the PRODEMOS platform[29]. In addition to the transition of the participants' end of the intervention into a mobile app,

For peer review only

adjustments were made to the platform in repeated cycles of interaction with end-users. In an iterative process, experiences, needs, and wishes from the target population and health coaches regarding the app and coach-support, gained through interviews and focus groups, served as a guideline for further development.

Participants randomised to the intervention condition will have access to an interactive smartphone application in their own language (English in the UK, Mandarin in China). The intervention app facilitates coach-supported self-management of seven dementia risk factors, including overweight, unhealthy diet, insufficient physical activity, smoking, hypertension, dyslipidaemia, and diabetes. Participants can set personal goals for lifestyle change, following the SMART (specific, measurable, achievable, realistic, time-bound) principle. Participants receive automated reminders to enter measurements (e.g. number of steps, blood pressure) for these goals, facilitating progress monitoring. The intervention participants will receive support from an experienced lifestyle coach, who is trained in motivational interviewing and works according to a coach protocol based on current guidelines for risk factor management. Regular training sessions in each country will enhance uniformity in coaching procedures, taking cultural differences into account. During the baseline visit, after randomisation, the coach discusses the participant's dementia risk profile and a first lifestyle goal will be set through the app. After the baseline visit, all communication between participant and coach will take place through the messaging functionality. Through the coach portal, the coach can view goals and measurements, send tailored education modules and videos, and can offer remote support to facilitate sustainable behaviour change.

Participants randomised to the control condition will have access to the control app, which is similar in appearance, but only contains education material, lacking interactive features and coach-support. During the baseline visit, control participants will receive concise feedback on their risk profile.

The PRODEMOS intervention in its current design is positioned as add-on to existing care.

For peer review only

<u>Primary and secondary outcomes</u>

Following a type II hybrid design, primary outcomes for effectiveness and implementation are equally important. The primary effectiveness outcome is the change in the Cardiovascular risk factors, Aging and Incidence of Dementia (CAIDE) dementia risk score between baseline and 18-months follow-up[30]. The main secondary effectiveness outcomes include the change in the individual modifiable components of the primary outcome, change in ten-year CVD risk, and cost-effectiveness. Clinical outcomes include incidence of mild cognitive impairment (MCI), dementia, CVD (stroke or myocardial infarction), and all-cause mortality. The operationalisation of all effectiveness outcomes is listed in table 2.

Implementation outcomes include acceptability, adoption, appropriateness, feasibility, fidelity, coverage, sustainability, and costs of the implementation. Outcomes will be assessed using quantitative and qualitative methods, both after three months and after the study. Qualitative studies using in-depth interviews with participants and coaches will focus on user-experiences, particularly with respect to barriers and facilitators for (sustained) platform use. All implementation outcomes and evaluation methods are shown in table 3.

Study logistics and data collection

The trial design is visualised in figure 2. All participants will receive one phone call and make three visits to a study venue during the study. Data is collected in electronic case report forms (eCRF) that are accessible through the assessor portal (figure 1).

Eligibility criteria that can be assessed remotely will be checked by a local research team member through the screening phone call. During the subsequent screening visit, informed consent will be obtained and final eligibility will be assessed by administering the Mini-Mental State Examination (MMSE), measuring blood pressure, BMI, waist circumference, total cholesterol (capillary blood sample in the UK; venous blood sample in China), and assessing physical activity, smoking behaviour, and a brief medical history. Weight will be measured with a calibrated scale, blood pressure will be measured twice with a calibrated, automated blood pressure device. Screening visits will be performed by (GP) nurses and local research team members specifically trained to perform these measurements, and will take place at the GP surgery or a nearby community venue. Standard Operating Procedures will be used to achieve uniform measurements within and between countries.

After the screening visit, all participants will fill in eight self-assessment questionnaires in the PRODEMOS app. These questionnaires will be used to assess secondary outcomes (table 2) and potential barriers for lifestyle behaviour change, which can inform coaches to tailor their coaching strategy. Seven of these questionnaires have been externally validated in both Western and Chinese populations[31-44]. Owing to obvious cultural differences, we decided to use two different diet questionnaires that were validated in the UK and Chinese population respectively[45 46].

The baseline visit will be conducted face-to-face by the health coach at the GP practice or local community venue. During this visit, self-assessment questionnaires are reviewed, relevant medical history and medication use are recorded, and participants are randomly assigned to one of the treatment conditions. Only intervention participants will set a first lifestyle goal together with the coach, according to their dementia risk profile.

All participants will receive periodic adverse event (AE) questionnaires in the app, assessing incident dementia, MCI, CVD, and diabetes. All self-reported outcomes will be verified with the participant's treating physician.

For peer review only

After 18 months, the questionnaires and all measurements performed during the screening- and baseline visit are repeated during the final visit.

Randomisation and blinding

After completion of the baseline assessments, participants will be individually randomised in a 1:1 ratio, stratified by country, using a central, computer-generated sequence. Participating cohabiting partners will be allocated to the same study condition. Complete blinding of participants is not possible, owing to the nature of the intervention. Participants will be informed that they will be randomised to one of two lifestyle apps, without further details. All outcome assessments will be done by an independent assessor unaware of treatment allocation.

Safety and privacy

Due to the nature of the intervention, serious AE's are unlikely to occur, and we consider the intervention low risk. A data safety and monitoring board is not installed.

Some precautions are taken to optimise participant safety. Firstly, regardless of their study allocation, participants will be referred to their GP or treating physician if deemed necessary based on their baseline or outcome parameters and local guidelines. Secondly, AE's will be monitored through three six-monthly questionnaires. Participants will receive notifications for these questionnaires on their smartphone and will receive reminders through e-mail (UK) or SMS (China). If the participant is not able to fill in the questionnaire, an informant can be contacted. A blinded researcher will, with explicit permission gained through the informed consent procedure, cross-check all reported AE's by consulting the participant's GP or treating physician. Thirdly, the PRODEMOS platform is built in accordance with the highest security requirements in healthcare. It complies with NEN 7510, the Health Insurance Portability and Accountability Act (HIPAA), ISO133485, and General Data Protection Regulation (GDPR). Data from the eCRFs and questionnaires will be exported in a pseudonymised format according to prevailing guidelines on good clinical practice in both countries. Only anonymised data will be exchanged between the UK, China, and the Netherlands. The exported data will be stored centrally on a protected server in the Netherlands, which is compatible with the highest standards of data management in medical research.

Protocol adjustments due to COVID-19 pandemic

As a result of the COVID-19 pandemic and related local research restrictions, certain adjustments have been made to the original study protocol as published on ISRCTN. Firstly, recruitment was planned to start in early 2020, but is now foreseen to start in January 2021. Second, as it is difficult to predict the development of the pandemic and associated restrictions, we have

For peer review only

slightly amended the study protocol to allow for flexible measurement procedures at baseline that can be operationalised in either one or two face-to-face visits, and for a flexible intervention duration of 12-18 months. However, we will strive for a follow-up period of 18 months in as many participants as possible.

Patient and Public Involvement

We have received valuable input into the design of the study and mHealth platform from interviews and focus groups with potential end-users in both countries. All patient facing material used in the UK has been reviewed by potential end-users and other stakeholders. Qualitative evaluations of the pilot study with research staff, coaches and patient participants were used to refine the intervention and study procedures.

For peer review only

Statistical analysis

Sample size

The CAIDE score will be used as primary effectiveness outcome. We decided to use a difference of 0.186 points on the CAIDE score between the average of both study groups as a minimum target threshold, because this difference was observed in the Prevention of Dementia by Intensive Vascular care (preDIVA) trial after two years (p=0.005; intervention group -0.290±1.47 SD, control group -0.104±1.36 SD). Attrition after two years of follow-up was 21% in this study[14]. With 80% power, a 0.05 two-sided significance level, accounting for 21% attrition, and a mean difference in change in CAIDE of 0.186, the required sample size is estimated to be 2319 participants. To allow for unexpected factors we raised this to 2400.

Data analysis

The effect on the CAIDE score will be analysed using linear mixed effects models according to the intention-to-treat principle, taking clustering within partner pairs and country into account by testing best fit for random intercept and/or slope. If needed, we will adjust for baseline imbalances and take clustering of the intervention within centre and/or coach into account. No imputation of the CAIDE score will be done for the primary analysis. In sensitivity analyses, we will use multiple imputation to assess the impact of missing items needed to calculate the CAIDE score, provided there are no indications that the variables are missing not at random (MNAR), and a per protocol analysis for those adherent to the intervention will be performed. Moreover, we will explore the interaction of intervention duration with the effect of the invention by adding an interaction term (intervention duration*randomisation group) to the main model. This will give insight into the potential additional intervention effect in participants with a follow-up time of less than 18 months.

Subgroup analyses will be performed for country, sex, age group, participants with(out) a history of CVD, the number of risk factors, willingness to change lifestyle at the start of the trial (assessed with one question during the baseline visit), participation with(out) a participating partner, having the same coach during the full length of the study, and the number of goals set. For all these factors, interaction terms will be included to test for between-subgroup differences in intervention effects.

The effect on the individual modifiable components of the CAIDE score and the 10-year CVD risk scores will be analysed using linear mixed effects models according to the intention to treat principle taking clustering within partner pairs and country into account. Self-assessment scales, which are mostly ordinal, will be regarded as linear scales, if there are at least four categories and the 'distance' between the categories can be regarded equal. Poisson regression or zero-inflated models

For peer review only

may be applied to distributions resembling count or zero-inflated data. The choice of the final model will be a compromise between optimal fit and interpretability of the results for a general clinical public.

Prevalence ratios will be used for self-assessment instruments with defined cut-offs for the presence or absence of a condition, e.g. ‘depressive symptoms’ for a GDS>5. For (clinical) dichotomous outcomes, such as incident CVD, dementia or mortality, Cox-proportional hazard models will be used with time since baseline as timescale. A sensitivity analysis will be performed using age as timescale.

The full analysis plan - including the health economic analysis plan entailing the cost-consequence analysis of the within trial results, the cost-effectiveness analyses, and the cost-utility analysis, and hypotheses for the subgroup analyses - is published on the ISRCTN website: http://www.isrctn.com/ISRCTN15986016.

For peer review only

Pilot study

Between December 2019 and March 2020, a six-week pilot study was conducted in the Brighton and Sussex area, UK. Since the main aim of the pilot study was to test study logistics and the functionality of the intervention app, participants were randomised in a 3:1 (intervention:control) ratio. An invitation letter was sent to 600 potentially eligible patients from two GPs. The response rate was 14.8% (n=89), of whom 21 participants (3.5%) could be included. Main reasons for exclusion were not living in the designated postal code area and having <2 dementia risk factors. Participants had a median age of 69 years (range 56-75), and 12 (57%) were male. Fifteen participants were allocated to the intervention group and six to the control group.

During the pilot study, 10 of 15 intervention participants set at least one goal (range 1–8 goals) through the app. Goals were set in five domains, including physical activity, healthy diet, body weight, blood pressure, and cholesterol. Six of ten participants entered goal-related measurements (range 2–243 measurements) to monitor their progress. All intervention participants used the chat functionality to consult the coach. In total 278 messages were sent back and forth, i.e. on average 3 messages per intervention participant per week.

The pilot study was evaluated through focus groups and individual interviews with participants and coaches. The main adjustments based on the participants' feedback included improvements to the chat functionality (the possibility to attach files, larger font size), simplification of the functionality to enter and view measurements, setting the first goal together with the coach, and more detailed instructions for app use through an instruction video and written manual. Based on feedback from the coaches, we improved the functionalities for population management in the coach portal, including an input screen to make notes about individual participants and a functionality to send education material to (groups of) participants.

A similar pilot study will be conducted in China, to test platform functionality and study logistics in all seven participating trial centres.

For peer review only

Discussion

In the PRODEMOS study we will investigate the implementation of a self-management mHealth intervention with remote coaching and its effect on dementia risk profiles over 18 months. We will target people aged 55-75 years with elevated dementia risk of low SES in the UK and of any SES in the Beijing and Tai'an cities in China, as these populations are usually not reached by preventive strategies and may benefit most. User data and qualitative analysis of our pilot study suggest that our mHealth application, after further adaptations to improve attractiveness and usability, is now ready to be studied in older adults who are interested in participating in a study on lifestyle change to lower their overall dementia risk.

The HATICE trial has shown that a coach-supported internet platform can improve cardiovascular risk factors in European elderly. Although we build on these previous experiences, the modality (i.e. app instead of internet platform) and target population are different. The resulting uncertainty that there would be a similar benefit of our intervention renders the use of a hybrid effectiveness-implementation design highly suitable[26].

Strengths

Chronic disease risk is largely affected by socioeconomic factors, including psychological, cultural, and economic characteristics, requiring preventive strategies that take these aspects into account[47]. In PRODEMOS we aim to support individuals by offering intensive human support through the app, and by aligning the intervention with the healthcare system. In order to eventually embed a complex prevention intervention into primary healthcare, it is crucial to involve and consult all stakeholders, such as GPs, specialised doctors, practice nurses, and end-users[48]. In the current hybrid effectiveness-implementation study we take some first steps to explore the possibilities and challenges for embedding the intervention in existing healthcare. This study will provide concrete evidence of the scale of the change that might be achieved for individuals at risk, whether and how this approach is taken up within diverse populations.

The PRODEMOS study is designed as one trial, recruiting participants in two different countries, increasing the external validity of the results. Overall, both countries will follow the same research protocol and highly similar standard operating procedures and will investigate similar interventions. Through semi-structured interviews among elderly in Beijing and the UK, we learned that needs and wishes regarding lifestyle behaviour change through mHealth are largely similar (manuscripts currently being drafted). Therefore, the Chinese and UK intervention will share the same functionalities and coaching procedures. Given obvious social, cultural, and healthcare-related differences, certain aspects of the study logistics, lifestyle support, and lay-out of the app had to be

For peer review only

culturally adjusted. In a pre-planned subgroup analysis, we will assess both effectiveness and implementation outcomes for both countries separately.

Limitations

The study design may yield some limitations. Firstly, the optimal age range for trials on dementia risk reduction is unknown[15]. There is a trade-off between potentially more effective treatments in midlife, and the chance to detect treatment effects on cognitive outcomes in late life[4]. As in the current study we are assessing both a dementia risk score and clinical outcomes, we have taken a pragmatic approach, targeting individuals aged 55-75 years[15].

Secondly, change in CAIDE dementia risk score is not easily translated into incidence of dementia. However, although not specifically designed as RCT outcome measure, the CAIDE score can detect change over time[49].

A third potential limitation is that, owing to the nature of the intervention, blinding of the participants is only partly possible. A certain degree of contamination might occur, especially in densely populated areas and in communities that live closely together. The study logistics and intervention are designed in such a way as to limit contact between participants after randomization.

Finally, the results of the baseline measurements will be revealed to all participants, potentially leading to treatment effects in both study conditions. Also, behaviour of participants and their treating physicians may change in both study conditions as a reaction to the awareness of being part of the study (Hawthorne-effect). Both mechanisms will perhaps mask (part of) the "true" contrast in dementia risk between the intervention and control condition.

For the planned health economic analyses we will rely on economic modelling, based on the intermediate outcomes reflecting risk of dementia and CVD and assumptions on their causality with clinical endpoints dementia and CVD, because the study is not designed nor powered to detect an effect on these clinical endpoints.

Conclusion

The high prevalence of dementia and lower provision of high-quality cardiovascular preventive care in LMIC and lower uptake of such programmes in western low SES populations require affordable and straightforward preventive strategies. If proven effective and implementable, our pragmatic smartphone intervention facilitates widespread use and reduction of dementia risk for hard-to-reach populations across the globe.

For peer review only

Table 1. Overview of in- and exclusion criteria.

Inclusion criteria

- Age ≥ 55 years ≤ 75 years
- Living in a postal code area ranked as equal to or less than the lowest 3rd decile of IMD[a]
- Good proficiency of the national language (English in UK, Mandarin in China)
- Possession of a smartphone
- ≥ 2 Dementia risk factors:
    - Insufficient physical activity (self-reported intermediate or vigorous activity of < 150 minutes per week)
    - Active smoking (self-reported use of any sort of tobacco in any quantity)
    - Depression:
        - Current diagnosis by specialist or GP or;
        - History of treatment for depression (i.e. drug therapy or psychotherapy)
    - Manifest cardiovascular disease, as diagnosed by specialist or GP
    - Diabetes mellitus:
        - Diagnosed by specialist or GP or;
        - Use of insulin or other blood glucose-lowering medication
    - Hypertension:
        - Diagnosed by specialist or GP or;
        - Use of blood pressure-lowering medication or;
        - Mean of baseline blood pressure measurements of ≥ 140 (systolic) or ≥ 90 (diastolic)
    - Obesity:
        - BMI ≥ 30 (UK), ≥ 28 (China) or;
        - Baseline waist circumference ≥ 102 cm (men in UK), 90 cm (men in China), 88 cm (women in UK), 85 cm (women in China)
    - Dyslipidemia:
        - Diagnosed by specialist or GP or;
        - Use of lipid-lowering medication or;
        - Baseline total cholesterol ≥ 5.0 mmol/L[a]

Exclusion criteria

- Manifest dementia, as diagnosed by specialist or GP
- MMSE < 24 (participants with ISCED level of > 1), MMSE <21 (participants with ISCED level of 1)
- Any condition expected to limit 18-months follow-up, including metastasized malignancy or other terminal illnesses
- Smartphone illiteracy, defined as not being able to send a message from a smartphone
- Visual impairment interfering with operation of a smartphone
- Participating in another RCT on behavior change
- Present severe alcohol or illicit drug abuse

[a] Applies only to participants in United Kingdom. BMI = Body Mass Index; GP = general practitioner; IMD = Index of Multiple Deprivation; ISCED = International Standard Classification of Education; MMSE = Mini Mental State examination; RCT = Randomised Controlled Trial; UK = United Kingdom.

For peer review only

Table 2. Effectiveness outcomes.

**Primary outcome**		
CAIDE score (range 0-15), which is comprised of and calculated from:		Points
Age	<47 years	0
	47-53	3
	>53	4
Education	≥10 years	0
	7-9 years	2
	<7 years	3
Gender	Female	0
	Male	1
Systolic blood pressure	≤140 mmHg	0
	>140 mmHg	2
BMI	≤30 kg/m^2	0
	>30 kg/m^2	2
Total cholesterol	≤6.5 mmol/L	0
	>6.5 mmol/L	2
Physical activity [a]	Yes	0
	No	1
**Secondary outcomes**		
Individual modifiable components of the CAIDE score[b]	Estimated 10-year cardiovascular risk	
Number of uncontrolled risk factors	LIBRA dementia risk score	
Active smoking	Number of hospital admissions	
Medication adherence	Diet [e]	
Number of drugs	Disability [f]	
Incident dementia[c]	Anxiety [g]	
Incident MCI[c]	Self-management [h]	
Incident cardiovascular disease[c,d]	Depressive symptoms [i]	
Incident diabetes[c]	Quality of life [j]	
All-cause mortality	Cost-effectiveness	

[a] assessed according to WHO standard for physical activity of at least 150 minutes per week; [b] physical activity assessed with the International Physical Activity Questionnaire Short Form; [c] self-reported and cross-checked with GP file; [d] Defined as myocardial infarction or stroke; [e] assessed with short-form food frequency questionnaire (UK) and China Kadoorie Biobank food frequency questionnaire (China); [f] assessed with the WHO Disability Assessment Schedule; [g] assessed with the Hospital Anxiety and Depression Scale – Anxiety; [h] assessed with the Partners In Health; [i] assessed with the Geriatric Depression Scale; [j] assessed with the ICEpop CAPability measure for Adults and EuroQol 5 dimensions 3 levels. BMI = Body Mass Index; CAIDE = Cardiovascular Risk Factors, Aging and Dementia; LIBRA = Lifestyle for Brain Health; MCI = Mild Cognitive Impairment; UK = United Kingdom.

For peer review only

Table 3. Summary of implementation research methods and outcomes.

Method	Outcome	Measurement	Population[a]	Timing of assessment
Quantitative	Coverage	(Non)response rates, comparison characteristics of participants with eligible population	Potential target population	At baseline
	Adoption	Utilisation, usage, and uptake	Intervention participants, coaches	At baseline
	Appropriateness	Short questionnaire of perceived fit or relevance in the target population and the coaches	Intervention participants, coaches	After 3 months and at study end
	Acceptability	Short questionnaire of agreeability, user-friendliness, credibility	Intervention participants, coaches	After 3 months and at study end
	Sustainability	Adherence, dropout	Intervention participants, dropouts[b]	Throughout the study
	Cost	Implementation costs	N.a.	N.a.
Qualitative	Feasibility	The extent to which the mHealth intervention can be carried out → practical and social barriers/facilitators	Intervention participants, dropouts[b], coaches	After 3 months and at study end
	Appropriateness	Perceived fit or relevance in the target population	Intervention participants, dropouts[b], coaches	After 3 months and at study end
	Acceptability	Agreeability, user-friendliness, credibility	Intervention participants, dropouts[b], coaches	After 3 months and at study end
	Fidelity	Degree to which the mHealth application is implemented compared to the original protocol	N.a.	After the study

[a] For all analyses a Chinese and UK population will be involved. [b] Study dropouts will be asked to participate in a short exit-interview. mHealth = mobile health. AE = adverse event.

For peer review only

Figure 1. Overview of the PRODEMOS platform and its functionalities

Legend: The control application has similar connections with the assessor portal and the researcher portal / database, but is not connected to the coach portal.

Figure 2. Trial design

Legend: AE = Adverse Event, UK = United Kingdom.

For peer review only

Dissemination
Results will be published in a peer-reviewed journal.

Author affiliations
[1] Department of General Practice, Amsterdam University Medical Centre, University of Amsterdam, Amsterdam, The Netherlands
[2]Department of Neurology, Amsterdam University Medical Centre, University of Amsterdam, Amsterdam, The Netherlands
[3]Beijing Key Laboratory of Clinical Epidemiology, School of Public Health, Capital Medical University, Beijing, China
[4]School of Medical and Health Sciences, Edith Cowan University, Perth, Australia
[5]INSERM/University of Toulouse UMR1027, Toulouse, France
[6]Department of Epidemiology and Public Health, Toulouse University Hospital, Toulouse, France
[7]Department of Public Health and Primary Care, Cambridge Institute of Public Health, University of Cambridge, Cambridge, United Kingdom
[8]Alzheimer Europe, Luxemburg City, Luxemburg
[9]Department of Primary Care and Public Health, Brighton and Sussex Medical School, Brighton, United Kingdom
[10]Vital Health Software, Ede, Netherlands
[11]Department of Public and Occupational Health, Amsterdam University Medical Centre, University of Amsterdam, Amsterdam, The Netherlands
[12]Alzheimer Centre Limburg, School for Mental Health and Neuroscience, Maastricht University Medical Centre+, Maastricht, the Netherlands
[13]Division of Neurogeriatrics, Department of Neurobiology, Care Sciences and Society, Karolinska Institutet, Stockholm, Sweden
[14]School of Public Health, Shandong First Medical University & Shandong Academy of Medical Science, Tai'an, Shandong, China
[15]Beijing Neurosurgical Institute, Beijing, China
[16]Centre for Cognitive Disorders, Beijing Geriatric Hospital, Beijing, China
[17] Department of Geriatrics, The Second Medical Centre & National Clinical Research Centre for Geriatric Diseases, Chinese PLA General Hospital, Beijing, China
[18]Fuzhou Comvee Network & Technology Co., Ltd, Fuzhou, China
[19]Department of Neurology, Xuanwu Hospital, Capital Medical University, Beijing, China
[20]Health Management Institute, The Second Medical Centre & National Clinical Research Centre for Geriatric Diseases, Chinese PLA General Hospital, Beijing, China
[21]Centre for Cognitive Neurology, Department of Neurology, Beijing Tiantan Hospital, Capital Medical University, Beijing, China
[22]Department of Public Health, Amsterdam University Medical Centre, University of Amsterdam, Amsterdam, The Netherlands
[23]Department of Neurology, Donders Institute for Brain, Behaviour and Cognition, Radboud University Medical Centre, Nijmegen, The Netherlands

Acknowledgements
The authors thank the European Union's Horizon 2020 research and innovation programme and National Key R & D Programme of China for the funding of the trial. The authors thank Lonneke van Vught for her substantial contribution in the early phase of the trial design. The authors thank Kevin Hekert, Louwrens Knulst, and Michiel van Dam for their substantial contribution in building the platform. The authors thank Jinxia Zhang, Xiaoyu Zhang, Mingyang Cao, Bin Jiang, Siqi Ge, Maolong Gao, Mo Li, Nayan Huang, Danning Li, Mingyue He, Weijiao Zhang, Huiying Guan, Jinghui Li, Yan Gong, Na Niu, Xiang Jia, Fei Wang, Jing Sun, Liyong Wu, Dan Li, Baoliang Sun, Hui Yuan, Guohua Wang, Xizhu Xu, Cancan Li, Wenran Zhang, Juan Du, and Libin Song for their contributions to the trial logistics. The authors thank all patient participants and other stakeholders who took part in PPI groups for their contribution to the platform and study design.

Author contributions
EE was responsible for the drafting of the manuscript. ER, EPMvC, WAvG, MHB, CB, and WW were responsible for the study conception. All other authors were responsible for the study design and critically revised the manuscript. All authors approved the final version of the manuscript.

Funding
This project has received funding from the European Union's Horizon 2020 research and innovation programme under grant agreement No 779238 and the National Key R&D Programme of China (2017YFE0118800).

Competing interests statement
We have no competing interests to declare.

Word count
3997

Ethics approval

The PRODEMOS trial is sponsored in the UK by the University of Cambridge and is granted ethical approval by the London – Brighton & Sussex Research Ethics Committee (REC Reference: 20/LO/01440). In China the trial is approved by the Medical Ethics Committees of Capital Medical University (CMU), Beijing Tiantan Hospital affiliated to CMU, Beijing Geriatric Hospital, the Chinese People's Liberation Army General Hospital, Taishan Medical University, and the Xuanwu Hospital affiliated to CMU.

For peer review only

Literature

1. Prince M. World Alzheimer Report 2015. The global impact of dementia. An analysis of prevalence, incidence, cost and trends. 2015
2. Yaffe K, Falvey C, Harris TB, et al. Effect of socioeconomic disparities on incidence of dementia among biracial older adults: prospective study. *BMJ* 2013;347:f7051. doi: 10.1136/bmj.f7051 [published Online First: 2013/12/21]
3. Livingston G, Huntley J, Sommerlad A, et al. Dementia prevention, intervention, and care: 2020 report of the Lancet Commission. *Lancet* 2020;396(10248):413-46. doi: 10.1016/S0140-6736(20)30367-6 [published Online First: 2020/08/03]
4. Qiu C, Winblad B, Fratiglioni L. The age-dependent relation of blood pressure to cognitive function and dementia. *Lancet Neurol* 2005;4(8):487-99. doi: 10.1016/S1474-4422(05)70141-1
5. Qiu C, Xu W, Fratiglioni L. Vascular and psychosocial factors in Alzheimer's disease: epidemiological evidence toward intervention. *J Alzheimers Dis* 2010;20(3):689-97. doi: 10.3233/JAD-2010-091663 [published Online First: 2010/02/26]
6. Barnes DE, Yaffe K. The projected effect of risk factor reduction on Alzheimer's disease prevalence. *Lancet Neurol* 2011;10(9):819-28. doi: 10.1016/S1474-4422(11)70072-2 [published Online First: 2011/07/22]
7. Kivipelto M, Mangialasche F, Ngandu T. Lifestyle interventions to prevent cognitive impairment, dementia and Alzheimer disease. *Nat Rev Neurol* 2018;14(11):653-66. doi: 10.1038/s41582-018-0070-3 [published Online First: 2018/10/07]
8. Andrieu S, Coley N, Lovestone S, et al. Prevention of sporadic Alzheimer's disease: lessons learned from clinical trials and future directions. *Lancet Neurol* 2015;14(9):926-44. doi: 10.1016/S1474-4422(15)00153-2 [published Online First: 2015/07/28]
9. van Middelaar T, van Vught LA, van Gool WA, et al. Blood pressure-lowering interventions to prevent dementia: a systematic review and meta-analysis. *J Hypertens* 2018;36(9):1780-87. doi: 10.1097/HJH.0000000000001829 [published Online First: 2018/06/22]
10. Sprint Mind Investigators for the SPRINT Research Group. Effect of Intensive vs Standard Blood Pressure Control on Probable Dementia: A Randomized Clinical Trial. *JAMA* 2019;321(6):553-61. doi: 10.1001/jama.2018.21442 [published Online First: 2019/01/29]
11. Hughes D, Judge C, Murphy R, et al. Association of Blood Pressure Lowering With Incident Dementia or Cognitive Impairment: A Systematic Review and Meta-analysis. *JAMA* 2020;323(19):1934-44. doi: 10.1001/jama.2020.4249 [published Online First: 2020/05/20]
12. Luchsinger JA, Reitz C, Honig LS, et al. Aggregation of vascular risk factors and risk of incident Alzheimer disease. *Neurology* 2005;65(4):545-51. doi: 10.1212/01.wnl.0000172914.08967.dc [published Online First: 2005/08/24]
13. Yaffe K. Metabolic syndrome and cognitive disorders: is the sum greater than its parts? *Alzheimer Dis Assoc Disord* 2007;21(2):167-71. doi: 10.1097/WAD.0b013e318065bfd6 [published Online First: 2007/06/05]
14. Moll van Charante EP, Richard E, Eurelings LS, et al. Effectiveness of a 6-year multidomain vascular care intervention to prevent dementia (preDIVA): a cluster-randomised controlled trial. *Lancet* 2016;388(10046):797-805. doi: 10.1016/S0140-6736(16)30950-3 [published Online First: 2016/07/31]
15. Richard E, Andrieu S, Solomon A, et al. Methodological challenges in designing dementia prevention trials - the European Dementia Prevention Initiative (EDPI). *J Neurol Sci* 2012;322(1-2):64-70. doi: 10.1016/j.jns.2012.06.012 [published Online First: 2012/07/24]
16. Coley N, Andrieu S, Gardette V, et al. Dementia prevention: methodological explanations for inconsistent results. *Epidemiol Rev* 2008;30:35-66. doi: 10.1093/epirev/mxn010 [published Online First: 2008/09/10]

For peer review only

17. Eggink E, Moll van Charante EP, van Gool WA, et al. A Population Perspective on Prevention of Dementia. *J Clin Med* 2019;8(6) doi: 10.3390/jcm8060834 [published Online First: 2019/06/20]
18. Beishuizen CR, Stephan BC, van Gool WA, et al. Web-Based Interventions Targeting Cardiovascular Risk Factors in Middle-Aged and Older People: A Systematic Review and Meta-Analysis. *J Med Internet Res* 2016;18(3):e55. doi: 10.2196/jmir.5218 [published Online First: 2016/03/13]
19. Richard E, Moll van Charante E, Hoevenaar-Blom MP, et al. Healthy ageing through internet counselling in the elderly (HATICE): a multinational, randomised controlled trial. 2019;1(8):e424-e34. doi: https://doi.org/10.1016/S2589-7500(19)30153-0
20. O'Dea S. Smartphone penetration rate by country 2018

2018 [Available from: https://www.statista.com/statistics/539395/smartphone-penetration-worldwide-by-country/ accessed 28-01-2021.
21. Statista. Do you personally use a smartphone?* - by socio-economic group 2019 [Available from: https://www.statista.com/statistics/300421/smartphone-usage-in-the-uk-by-socio-economic-group/ accessed 28-01-2021.
22. Smartphone penetration rate in China from 2015 to 2023 [Available from: https://www.statista.com/statistics/321482/smartphone-user-penetration-in-china/ accessed 28-01-2021.
23. Coorey GM, Neubeck L, Mulley J, et al. Effectiveness, acceptability and usefulness of mobile applications for cardiovascular disease self-management: Systematic review with meta-synthesis of quantitative and qualitative data. *Eur J Prev Cardiol* 2018;25(5):505-21. doi: 10.1177/2047487317750913 [published Online First: 2018/01/10]
24. Piette JD, List J, Rana GK, et al. Mobile Health Devices as Tools for Worldwide Cardiovascular Risk Reduction and Disease Management. *Circulation* 2015;132(21):2012-27. doi: 10.1161/CIRCULATIONAHA.114.008723 [published Online First: 2015/11/26]
25. Curran GM, Bauer M, Mittman B, et al. Effectiveness-implementation hybrid designs: combining elements of clinical effectiveness and implementation research to enhance public health impact. *Med Care* 2012;50(3):217-26. doi: 10.1097/MLR.0b013e3182408812 [published Online First: 2012/02/09]
26. Landes SJ, McBain SA, Curran GM. An introduction to effectiveness-implementation hybrid designs. *Psychiatry Res* 2019;280:112513. doi: 10.1016/j.psychres.2019.112513 [published Online First: 2019/08/23]
27. Payne RA, Abel GA. UK indices of multiple deprivation – a way to make comparisons across constituent countries easier. *Health Statistics Quarterly* 2012;53
28. Wang H, Zhai F. Programme and policy options for preventing obesity in China. *Obes Rev* 2013;14 Suppl 2:134-40. doi: 10.1111/obr.12106 [published Online First: 2013/10/10]
29. Jongstra S, Beishuizen C, Andrieu S, et al. Development and Validation of an Interactive Internet Platform for Older People: The Healthy Ageing Through Internet Counselling in the Elderly Study. *Telemed J E Health* 2017;23(2):96-104. doi: 10.1089/tmj.2016.0066 [published Online First: 2016/07/28]
30. Kivipelto M, Ngandu T, Laatikainen T, et al. Risk score for the prediction of dementia risk in 20 years among middle aged people: a longitudinal, population-based study. *Lancet Neurol* 2006;5(9):735-41. doi: 10.1016/S1474-4422(06)70537-3 [published Online First: 2006/08/18]
31. Wang HM, Patrick DL, Edwards TC, et al. Validation of the EQ-5D in a general population sample in urban China. *Qual Life Res* 2012;21(1):155-60. doi: 10.1007/s11136-011-9915-6 [published Online First: 2011/04/21]
32. Deng HB, Macfarlane DJ, Thomas GN, et al. Reliability and validity of the IPAQ-Chinese: the Guangzhou Biobank Cohort study. *Med Sci Sports Exerc* 2008;40(2):303-7. doi: 10.1249/mss.0b013e31815b0db5 [published Online First: 2008/01/19]

For peer review only

33. Tang C, Xiong Y, Wu H, et al. Adaptation and assessments of the Chinese version of the ICECAP-A measurement. *Health Qual Life Outcomes* 2018;16(1):45. doi: 10.1186/s12955-018-0865-3 [published Online First: 2018/03/14]
34. Üstün T, Kostanjsek N, Chatterji S, et al. Measuring Health and Disability Manual for WHO disability assessment schedule WHODAS 2.0: World Health Organization, 2010.
35. Xie Z, Lv X, Hu Y, et al. Development and validation of the geriatric depression inventory in Chinese culture. *Int Psychogeriatr* 2015;27(9):1505-11. doi: 10.1017/S1041610215000162 [published Online First: 2015/02/24]
36. Xiaofei Z, Hui F, Lawn S, et al. Translation and initial psychometric evaluation of the Chinese version of the partners in health scale. *Biomedical research* 2017;28(16):7322-29.
37. Yang Y, Ding R, Hu D, et al. Reliability and validity of a Chinese version of the HADS for screening depression and anxiety in psycho-cardiological outpatients. *Compr Psychiatry* 2014;55(1):215-20. doi: 10.1016/j.comppsych.2013.08.012 [published Online First: 2013/11/10]
38. EuroQol G. EuroQol--a new facility for the measurement of health-related quality of life. *Health Policy* 1990;16(3):199-208. doi: 10.1016/0168-8510(90)90421-9 [published Online First: 1990/11/05]
39. Yesavage JA, Brink TL, Rose TL, et al. Development and validation of a geriatric depression screening scale: a preliminary report. *J Psychiatr Res* 1982;17(1):37-49. doi: 10.1016/0022-3956(82)90033-4 [published Online First: 1982/01/01]
40. Al-Janabi H, Flynn TN, Coast J. Development of a self-report measure of capability wellbeing for adults: the ICECAP-A. *Qual Life Res* 2012;21(1):167-76. doi: 10.1007/s11136-011-9927-2 [published Online First: 2011/05/21]
41. Al-Janabi H, Peters TJ, Brazier J, et al. An investigation of the construct validity of the ICECAP-A capability measure. *Qual Life Res* 2013;22(7):1831-40. doi: 10.1007/s11136-012-0293-5 [published Online First: 2012/10/23]
42. Reece M, Collins J, Markwick M, et al. The Partners in Health scale: The development and psychometric properties of a generic assessment scale for chronic condition self-management. *Australian Journal of Primary Health* 2003;9:41-52.
43. Craig CL, Marshall AL, Sjostrom M, et al. International physical activity questionnaire: 12-country reliability and validity. *Med Sci Sports Exerc* 2003;35(8):1381-95. doi: 10.1249/01.MSS.0000078924.61453.FB [published Online First: 2003/08/06]
44. Bjelland I, Dahl AA, Haug TT, et al. The validity of the Hospital Anxiety and Depression Scale. An updated literature review. *J Psychosom Res* 2002;52(2):69-77. doi: 10.1016/s0022-3999(01)00296-3 [published Online First: 2002/02/08]
45. Cleghorn CL, Harrison RA, Ransley JK, et al. Can a dietary quality score derived from a short-form FFQ assess dietary quality in UK adult population surveys? *Public Health Nutr* 2016;19(16):2915-23. doi: 10.1017/S1368980016001099 [published Online First: 2016/05/18]
46. Zhu N, Yu C, Guo Y, et al. Adherence to a healthy lifestyle and all-cause and cause-specific mortality in Chinese adults: a 10-year prospective study of 0.5 million people. *Int J Behav Nutr Phys Act* 2019;16(1):98. doi: 10.1186/s12966-019-0860-z [published Online First: 2019/11/07]
47. Havranek EP, Mujahid MS, Barr DA, et al. Social Determinants of Risk and Outcomes for Cardiovascular Disease: A Scientific Statement From the American Heart Association. *Circulation* 2015;132(9):873-98. doi: 10.1161/CIR.0000000000000228 [published Online First: 2015/08/05]
48. Volker N, Williams LT, Davey RC, et al. Implementation of cardiovascular disease prevention in primary health care: enhancing understanding using normalisation process theory. *BMC Fam Pract* 2017;18(1):28. doi: 10.1186/s12875-017-0580-x [published Online First: 2017/02/27]
49. Coley N, Hoevenaar-Blom MP, van Dalen JW, et al. Dementia risk scores as surrogate outcomes for lifestyle-based multidomain prevention trials-rationale, preliminary evidence and

For peer review only

challenges. *Alzheimers Dement* 2020 doi: 10.1002/alz.12169 [published Online First: 2020/08/18]

For peer review only

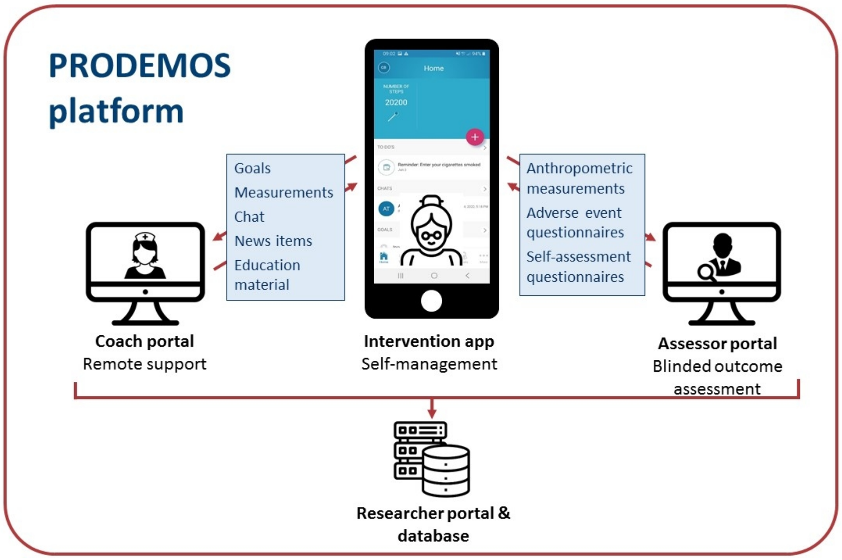

Caption: Figure 1. Overview of the PRODEMOS platform and its functionalities
Legend: The control application has similar connections with the assessor portal and the researcher portal / database, but is not connected to the coach portal.

338x190mm (96 x 96 DPI)

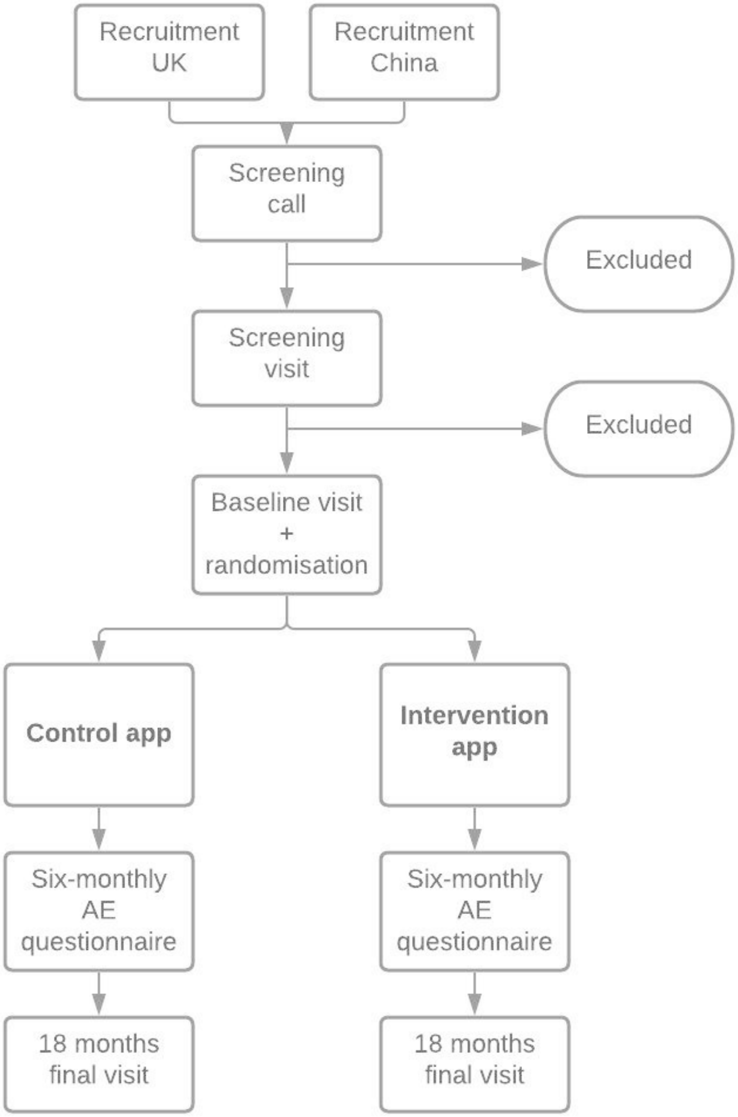

Capture: Figure 2. Trial design

Legend: AE = Adverse Event, UK = United Kingdom.

111x161mm (160 x 160 DPI)

Reporting checklist for protocol of a clinical trial.

Based on the SPIRIT guidelines.

Instructions to authors

Complete this checklist by entering the page numbers from your manuscript where readers will find each of the items listed below.

Your article may not currently address all the items on the checklist. Please modify your text to include the missing information. If you are certain that an item does not apply, please write "n/a" and provide a short explanation.

Upload your completed checklist as an extra file when you submit to a journal.

In your methods section, say that you used the SPIRITreporting guidelines, and cite them as:

Chan A-W, Tetzlaff JM, Gøtzsche PC, Altman DG, Mann H, Berlin J, Dickersin K, Hróbjartsson A, Schulz KF, Parulekar WR, Krleža-Jerić K, Laupacis A, Moher D. SPIRIT 2013 Explanation and Elaboration: Guidance for protocols of clinical trials. BMJ. 2013;346:e7586

		Reporting Item	Page Number
**Administrative information**			
Title	#1	Descriptive title identifying the study design, population, interventions, and, if applicable, trial acronym	1
Trial registration	#2a	Trial identifier and registry name. If not yet registered, name of intended registry	2
Trial registration: data set	#2b	All items from the World Health Organization Trial Registration Data Set	n/a
Protocol version	#3	Date and version identifier	n/a
Funding	#4	Sources and types of financial, material, and other support	21
Roles and responsibilities: contributorship	#5a	Names, affiliations, and roles of protocol contributors	21

For peer review only

Roles and responsibilities: sponsor contact information	#5b	Name and contact information for the trial sponsor	22
Roles and responsibilities: sponsor and funder	#5c	Role of study sponsor and funders, if any, in study design; collection, management, analysis, and interpretation of data; writing of the report; and the decision to submit the report for publication, including whether they will have ultimate authority over any of these activities	n/a
Roles and responsibilities: committees	#5d	Composition, roles, and responsibilities of the coordinating centre, steering committee, endpoint adjudication committee, data management team, and other individuals or groups overseeing the trial, if applicable (see Item 21a for data monitoring committee)	6
**Introduction**			
Background and rationale	#6a	Description of research question and justification for undertaking the trial, including summary of relevant studies (published and unpublished) examining benefits and harms for each intervention	4
Background and rationale: choice of comparators	#6b	Explanation for choice of comparators	4
Objectives	#7	Specific objectives or hypotheses	5
Trial design	#8	Description of trial design including type of trial (eg, parallel group, crossover, factorial, single group), allocation ratio, and framework (eg, superiority, equivalence, non-inferiority, exploratory)	6
**Methods: Participants, interventions, and outcomes**			
Study setting	#9	Description of study settings (eg, community clinic, academic hospital) and list of countries where data will be collected. Reference to where list of study sites can be obtained	6,8
Eligibility criteria	#10	Inclusion and exclusion criteria for participants. If applicable, eligibility criteria for study centres and individuals who will	5

For peer review only

		perform the interventions (eg, surgeons, psychotherapists)	
Interventions: description	#11a	Interventions for each group with sufficient detail to allow replication, including how and when they will be administered	6,7
Interventions: modifications	#11b	Criteria for discontinuing or modifying allocated interventions for a given trial participant (eg, drug dose change in response to harms, participant request, or improving / worsening disease)	n/a
Interventions: adherance	#11c	Strategies to improve adherence to intervention protocols, and any procedures for monitoring adherence (eg, drug tablet return; laboratory tests)	6,7
Interventions: concomitant care	#11d	Relevant concomitant care and interventions that are permitted or prohibited during the trial	7
Outcomes	#12	Primary, secondary, and other outcomes, including the specific measurement variable (eg, systolic blood pressure), analysis metric (eg, change from baseline, final value, time to event), method of aggregation (eg, median, proportion), and time point for each outcome. Explanation of the clinical relevance of chosen efficacy and harm outcomes is strongly recommended	7-8
Participant timeline	#13	Time schedule of enrolment, interventions (including any run-ins and washouts), assessments, and visits for participants. A schematic diagram is highly recommended (see Figure)	20
Sample size	#14	Estimated number of participants needed to achieve study objectives and how it was determined, including clinical and statistical assumptions supporting any sample size calculations	10
Recruitment	#15	Strategies for achieving adequate participant enrolment to reach target sample size	5
**Methods: Assignment of interventions (for controlled trials)**			
Allocation: sequence generation	#16a	Method of generating the allocation sequence (eg, computer-generated random numbers), and list of any factors for stratification. To reduce predictability of a random sequence, details of any planned restriction (eg, blocking) should be provided in a separate document that is unavailable to those who enrol participants or assign interventions	9

For peer review only

Allocation concealment mechanism	#16b	Mechanism of implementing the allocation sequence (eg, central telephone; sequentially numbered, opaque, sealed envelopes), describing any steps to conceal the sequence until interventions are assigned	8
Allocation: implementation	#16c	Who will generate the allocation sequence, who will enrol participants, and who will assign participants to interventions	9
Blinding (masking)	#17a	Who will be blinded after assignment to interventions (eg, trial participants, care providers, outcome assessors, data analysts), and how	9
Blinding (masking): emergency unblinding	#17b	If blinded, circumstances under which unblinding is permissible, and procedure for revealing a participant's allocated intervention during the trial	n/a
**Methods: Data collection, management, and analysis**			
Data collection plan	#18a	Plans for assessment and collection of outcome, baseline, and other trial data, including any related processes to promote data quality (eg, duplicate measurements, training of assessors) and a description of study instruments (eg, questionnaires, laboratory tests) along with their reliability and validity, if known. Reference to where data collection forms can be found, if not in the protocol	7-9, 16, 17
Data collection plan: retention	#18b	Plans to promote participant retention and complete follow-up, including list of any outcome data to be collected for participants who discontinue or deviate from intervention protocols	n/a
Data management	#19	Plans for data entry, coding, security, and storage, including any related processes to promote data quality (eg, double data entry; range checks for data values). Reference to where details of data management procedures can be found, if not in the protocol	9
Statistics: outcomes	#20a	Statistical methods for analysing primary and secondary outcomes. Reference to where other details of the statistical analysis plan can be found, if not in the protocol	9,10
Statistics: additional analyses	#20b	Methods for any additional analyses (eg, subgroup and adjusted analyses)	9,10

For peer review only

Statistics: analysis population and missing data	#20c	Definition of analysis population relating to protocol non-adherence (eg, as randomised analysis), and any statistical methods to handle missing data (eg, multiple imputation)	9,10
**Methods: Monitoring**			
Data monitoring: formal committee	#21a	Composition of data monitoring committee (DMC); summary of its role and reporting structure; statement of whether it is independent from the sponsor and competing interests; and reference to where further details about its charter can be found, if not in the protocol. Alternatively, an explanation of why a DMC is not needed	n/a
Data monitoring: interim analysis	#21b	Description of any interim analyses and stopping guidelines, including who will have access to these interim results and make the final decision to terminate the trial	n/a
Harms	#22	Plans for collecting, assessing, reporting, and managing solicited and spontaneously reported adverse events and other unintended effects of trial interventions or trial conduct	9
Auditing	#23	Frequency and procedures for auditing trial conduct, if any, and whether the process will be independent from investigators and the sponsor	n/a
**Ethics and dissemination**			
Research ethics approval	#24	Plans for seeking research ethics committee / institutional review board (REC / IRB) approval	22
Protocol amendments	#25	Plans for communicating important protocol modifications (eg, changes to eligibility criteria, outcomes, analyses) to relevant parties (eg, investigators, REC / IRBs, trial participants, trial registries, journals, regulators)	n/a
Consent or assent	#26a	Who will obtain informed consent or assent from potential trial participants or authorised surrogates, and how (see Item 32)	8
Consent or assent: ancillary studies	#26b	Additional consent provisions for collection and use of participant data and biological specimens in ancillary studies, if applicable	n/a
Confidentiality	#27	How personal information about potential and enrolled participants will be collected, shared, and maintained in order to protect confidentiality before, during, and after the trial	9

For peer review only

Declaration of interests	#28	Financial and other competing interests for principal investigators for the overall trial and each study site	21
Data access	#29	Statement of who will have access to the final trial dataset, and disclosure of contractual agreements that limit such access for investigators	6,9
Ancillary and post trial care	#30	Provisions, if any, for ancillary and post-trial care, and for compensation to those who suffer harm from trial participation	n/a
Dissemination policy: trial results	#31a	Plans for investigators and sponsor to communicate trial results to participants, healthcare professionals, the public, and other relevant groups (eg, via publication, reporting in results databases, or other data sharing arrangements), including any publication restrictions	21
Dissemination policy: authorship	#31b	Authorship eligibility guidelines and any intended use of professional writers	21
Dissemination policy: reproducible research	#31c	Plans, if any, for granting public access to the full protocol, participant-level dataset, and statistical code	n/a
**Appendices**			
Informed consent materials	#32	Model consent form and other related documentation given to participants and authorised surrogates	n/a
Biological specimens	#33	Plans for collection, laboratory evaluation, and storage of biological specimens for genetic or molecular analysis in the current trial and for future use in ancillary studies, if applicable	n/a

Notes:

- 18a: 7-9, 16, 17 The SPIRIT checklist is distributed under the terms of the Creative Commons Attribution License CC-BY-ND 3.0. This checklist was completed on 29. January 2021 using https://www.goodreports.org/, a tool made by the EQUATOR Network in collaboration with Penelope.ai

For peer review only

BMJ Open

Prevention of Dementia using Mobile Phone Applications (PRODEMOS): Protocol for an international randomised controlled trial.

Journal:	*BMJ Open*
Manuscript ID	bmjopen-2021-049762.R1
Article Type:	Protocol
Date Submitted by the Author:	29-Apr-2021
Complete List of Authors:	Eggink, Esmé; Amsterdam UMC Locatie AMC, Department of General Practice<br>Hafdi, Melanie; Amsterdam UMC Locatie AMC, Department of Neurology<br>Hoevenaar-Blom, Marieke ; Amsterdam UMC Locatie AMC, Department of Neurology<br>Song, Manshu; Capital Medical University School of Public Health, Beijing Key Laboratory of Clinical Epidemiology; Edith Cowan University School of Medical and Health Sciences<br>Andrieu, Sandrine; INSERM-University of Toulouse UMR1027; Toulouse University Hospital, Department of Epidemiology and Public Health<br>Barnes, Linda E; University of Cambridge, Cambridge Public Health<br>Birck, Cindy; Alzheimer Europe<br>Brooks, Rachael L; University of Cambridge, Cambridge Public Health<br>Coley, Nicola; INSERM-University of Toulouse UMR1027; Toulouse University Hospital, Department of Epidemiology and Public Health<br>Ford, Elizabeth; Brighton and Sussex Medical School, Department of Primary Care and Public Health<br>Georges, Jean; Alzheimer Europe<br>van der Groep, Abraham; Vital Health Software<br>van Gool, Willem A; Amsterdam UMC Locatie AMC, Department of Public and Occupational Health<br>Handels, Ron; School for Mental Health and Neuroscience, Maastricht University Medical Centre+, Alzheimer Centre Limburg; Karolinska Institutet, Division of Neurogeriatrics, Department of Neurobiology, Care Science and Society<br>HOU, Haifeng; Edith Cowan University School of Medical and Health Sciences; Shandong First Medical University & Shandong Academy of Medical Science, School of Public Health<br>Li, Dong; Shandong First Medical University & Shandong Academy of Medical Science, School of Public Health<br>Liu, Hongmei; Beijing Neurosurgical Institute<br>Lyu, Jihui; Beijing Geriatric Hospital, Center for Cognitive Disorders<br>van Marwijk, Harm; Brighton and Sussex Medical School, Department of Primary Care and Public Health<br>van der Meijden, Mark; Vital Health Software<br>Niu, Yixuan; The Second Medical Centre & National Clinical Research Centre for Geriatric Diseases, Chinese PLA General Hospital, Department of Geriatrics<br>Sadhwani, Shanu; Brighton and Sussex Medical School, Department of

	Primary Care and Public Health<br>Wang, Wenzhi; Beijing Neurosurgical Institute<br>Wang, Youxin; Capital Medical University School of Public Health, Beijing Key Laboratory of Clinical Epidemiology; Edith Cowan University School of Medical and Health Sciences<br>Wimo, Anders; Karolinska Institutet, Division of Neurogeriatrics, Department of Neurobiology, Care Sciences and Society<br>Ye, Xiaoyan; Fuzhou Comvee Network & Technology Co., Ltd<br>Yu, Yueyi; Xuanwu Hospital Capital Capital Medical University, Department of Neurology<br>Zeng, Qiang; The Second Medical Centre & National Clinical Research Centre for Geriatric Diseases, Chinese PLA General Hospital, Health Management Institute<br>Zhang, Wei; Beijing Tiantan Hospital, Capital Medical University, Centre for Cognitive Neurology, Department of Neurology<br>Wang, Wei; Capital Medical University School of Public Health, Beijing Key Laboratory of Clinical Epidemiology; Edith Cowan University School of Medical and Health Sciences<br>Brayne, Carol; University of Cambridge, Cambridge Public Health<br>Moll van Charante, Eric P; Amsterdam UMC Locatie AMC, Department of General Practice; Amsterdam UMC Locatie AMC, Department of Public and Occupational Health<br>Richard, Edo; Amsterdam UMC Locatie AMC, Department of Public and Occupational Health; Radboud University Donders Institute for Brain Cognition and Behaviour, Department of Neurology
**Primary Subject Heading**:	Public health
Secondary Subject Heading:	Cardiovascular medicine, Neurology
Keywords:	Dementia < NEUROLOGY, Adult neurology < NEUROLOGY, PUBLIC HEALTH

SCHOLARONE™
Manuscripts

I, the Submitting Author has the right to grant and does grant on behalf of all authors of the Work (as defined in the below author licence), an exclusive licence and/or a non-exclusive licence for contributions from authors who are: i) UK Crown employees; ii) where BMJ has agreed a CC-BY licence shall apply, and/or iii) in accordance with the terms applicable for US Federal Government officers or employees acting as part of their official duties; on a worldwide, perpetual, irrevocable, royalty-free basis to BMJ Publishing Group Ltd ("BMJ") its licensees and where the relevant Journal is co-owned by BMJ to the co-owners of the Journal, to publish the Work in this journal and any other BMJ products and to exploit all rights, as set out in our licence.

The Submitting Author accepts and understands that any supply made under these terms is made by BMJ to the Submitting Author unless you are acting as an employee on behalf of your employer or a postgraduate student of an affiliated institution which is paying any applicable article publishing charge ("APC") for Open Access articles. Where the Submitting Author wishes to make the Work available on an Open Access basis (and intends to pay the relevant APC), the terms of reuse of such Open Access shall be governed by a Creative Commons licence – details of these licences and which Creative Commons licence will apply to this Work are set out in our licence referred to above.

Other than as permitted in any relevant BMJ Author's Self Archiving Policies, I confirm this Work has not been accepted for publication elsewhere, is not being considered for publication elsewhere and does not duplicate material already published. I confirm all authors consent to publication of this Work and authorise the granting of this licence.

For peer review only

Protocol article

Prevention of Dementia using Mobile Phone Applications (PRODEMOS): protocol for an international randomised controlled trial

Esmé Eggink[1], Melanie Hafdi[2], Marieke P Hoevenaar-Blom[2], Manshu Song[3,4], Sandrine Andrieu[5,6], Linda E Barnes[7], Cindy Birck[8], Rachael L Brooks[7], Nicola Coley[5,6], Elizabeth Ford[9], Jean Georges[8], Abraham van der Groep[10], Willem A van Gool[11], Ron LH Handels[12,13], Haifeng Hou[4,14], Dong Li[14], Hongmei Liu[15], Jihui Lyu[16], Harm WJ van Marwijk[9], Mark van der Meijden[10], Yixuan Niu[17], Shanu Sadhwani[9], Wenzhi Wang[15], Youxin Wang[3,4], Anders Wimo[13], Xiaoyan Ye[18], Yueyi Yu[19], Qiang Zeng[20], Wei Zhang[21], Wei Wang[3,4,14]*, Carol Brayne[7]*, Eric P Moll van Charante[1,11]*, Edo Richard[11,22]*#

*Shared last authors

#Corresponding author:
Dr. Edo Richard
Meibergdreef 9, 1105 AZ Amsterdam
e.richard@amsterdamumc.nl
0205669111

Author affiliations

[1] Department of General Practice, Amsterdam University Medical Centre, University of Amsterdam, Amsterdam, The Netherlands
[2]Department of Neurology, Amsterdam University Medical Centre, University of Amsterdam, Amsterdam, The Netherlands
[3]Beijing Key Laboratory of Clinical Epidemiology, School of Public Health, Capital Medical University, Beijing, China
[4]School of Medical and Health Sciences, Edith Cowan University, Perth, Australia
[5]INSERM/University of Toulouse UMR1027, Toulouse, France
[6]Department of Epidemiology and Public Health, Toulouse University Hospital, Toulouse, France
[7]Cambridge Public Health, University of Cambridge, Cambridge, United Kingdom
[8]Alzheimer Europe, Luxemburg City, Luxemburg
[9]Department of Primary Care and Public Health, Brighton and Sussex Medical School, Brighton, United Kingdom
[10]Vital Health Software, Ede, Netherlands
[11]Department of Public and Occupational Health, Amsterdam University Medical Centre, University of Amsterdam, Amsterdam, The Netherlands
[12]Alzheimer Centre Limburg, School for Mental Health and Neuroscience, Maastricht University Medical Centre+, Maastricht, the Netherlands
[13]Division of Neurogeriatrics, Department of Neurobiology, Care Sciences and Society, Karolinska Institutet, Stockholm, Sweden
[14]School of Public Health, Shandong First Medical University & Shandong Academy of Medical Science, Tai'an, Shandong, China
[15]Beijing Neurosurgical Institute, Beijing, China
[16]Centre for Cognitive Disorders, Beijing Geriatric Hospital, Beijing, China
[17] Department of Geriatrics, The Second Medical Centre & National Clinical Research Centre for Geriatric Diseases, Chinese PLA General Hospital, Beijing, China
[18]Fuzhou Comvee Network & Technology Co., Ltd, Fuzhou, China
[19]Department of Neurology, Xuanwu Hospital, Capital Medical University, Beijing, China
[20]Health Management Institute, The Second Medical Centre & National Clinical Research Centre for Geriatric Diseases, Chinese PLA General Hospital, Beijing, China
[21]Centre for Cognitive Neurology, Department of Neurology, Beijing Tiantan Hospital, Capital Medical University, Beijing, China
[22]Department of Neurology, Donders Institute for Brain, Behaviour and Cognition, Radboud University Medical Centre, Nijmegen, The Netherlands

Key words: Dementia; prevention; lifestyle intervention; mobile health
Word count: 4000 (excluding title page, abstract and strength and limitations section)

For peer review only

Abstract

Introduction Profiles of high risk for future dementia are well understood and are likely to concern mostly those in low- and middle-income countries, and people at greater disadvantage in high-income countries. Approximately 30-40% of dementia cases have been estimated to be attributed to modifiable risk factors, including hypertension, smoking, and sedentary lifestyle. Tailored interventions targeting these risk factors can potentially prevent or delay the onset of dementia. Mobile Health (mHealth) improves accessibility of such prevention strategies in hard-to-reach populations, while at the same time tailoring such approaches. In the current study we will investigate the effectiveness and implementation of a coach-supported mHealth intervention, targeting dementia risk factors, to reduce dementia risk.

Methods and analysis The PRODEMOS RCT will follow an effectiveness-implementation hybrid design, taking place in the United Kingdom (UK) and China. People are eligible if they are 55-75 years, of low socioeconomic status (UK) or from the general population (China), have ≥2 dementia risk factors, and own a smartphone. 2400 participants will be randomised to either a coach-supported, interactive mHealth platform, facilitating self-management of dementia risk factors, or a static control platform. The intervention and follow-up period will be 18 months. The primary effectiveness outcome is change in the previously validated CAIDE dementia risk score. Main secondary outcomes include improvement of individual risk factors and cost-effectiveness. Implementation outcomes include acceptability, adoption, feasibility, and sustainability of the intervention.

Ethics and dissemination The PRODEMOS trial is sponsored in the UK by the University of Cambridge and is granted ethical approval by the London–Brighton&Sussex Research Ethics Committee (REC Reference:20/LO/01440). In China, the trial is approved by the Medical Ethics Committees of Capital Medical University (CMU), Beijing Tiantan Hospital, Beijing Geriatric Hospital, Chinese People's Liberation Army General Hospital, Taishan Medical University, and Xuanwu Hospital. Results will be published in a peer-reviewed journal.

Trial registration number: ISRCTN15986016

For peer review only - http://bmjopen.bmj.com/site/about/guidelines.xhtml

Strengths and limitations of this study

- The coach-supported mHealth intervention builds on the previously developed HATICE electronic health (eHealth) platform, which was shown to improve cardiovascular risk of elderly in the Netherlands, France, and Finland.

- The main strengths of the present study are: testing the approach in populations with low socioeconomic status or different cultural settings and measuring whether sustained involvement of end-users can be achieved, facilitating cultural adaptation of the application.

- Limitations of this study include the impossibility to completely blinding the participants, potentially leading to contamination, and the challenge to sufficiently engage the hard-to-reach target population.

For peer review only

Introduction

With global ageing the prevalence of dementia is expected to increase to over 130 million in 2050, especially in low- and middle-income countries (LMIC) and in people from low socioeconomic status (SES) background in high-income countries (HIC)[1 2]. Strategies need to be developed that aim to reduce the risk of dementia – many of which will be at community and population level, but those that are individually based must be effective, affordable, and easily implementable across various healthcare settings.

Up to 40% of dementia cases are estimated to be attributable to potentially modifiable risk factors[3], of which 10-20% are cardiovascular risk factors including hypertension, midlife obesity, dyslipidaemia, diabetes, smoking, and physical inactivity[4-6]. To date, intervention studies aiming to reduce dementia risk by targeting one or more of these risk factors have shown inconsistent results[7 8]. Results from randomised controlled trial (RCTs) on blood pressure lowering treatment have suggested a beneficial effect on dementia risk, although not consistently and convincingly[9-11]. Since the presence of multiple risk factors may pose an additive or even synergistic effect on dementia risk[12 13], targeting several risk factors simultaneously may be more effective. The only study to date designed to address this question using dementia as primary outcome did not show a statistically significant effect after 6-8 years of intervention, although subgroup analysis suggested benefit for those with untreated hypertension at baseline[14].

A considerable challenge when designing a dementia prevention trial is the time lag between the optimal timing of the intervention and the onset of dementia. Using incident dementia as primary outcome requires large sample sizes and/or long follow-up periods to reach statistical power[15 16]. Dementia risk scores could be used as a proxy, especially in trials with follow-up periods up to several years. Another challenge, possibly explaining the neutral results of intervention studies so far, is the small window for risk factor improvement given a background of high-quality cardiovascular risk management in HIC where these studies were performed[17]. This lends further support for targeting people in LMIC and low SES populations in HIC.

Digital health interventions have the potential to improve cardiovascular risk factors in middle-age and beyond, especially when offered with human coaching (blended-care)[18]. In the Healthy Ageing Through Internet Counselling in the Elderly (HATICE) trial, we recently demonstrated that a coach-supported internet intervention facilitating self-management of cardiovascular risk factors can reduce older adults' cardiovascular risk over a static control platform, both in high and low socioeconomic participant subgroups[19]. Currently, digital health interventions are increasingly offered through smartphones. Smartphone penetration rates are especially high in HIC[20], also among people with low SES. In 2018, 67% of people with the lowest SES in UK owned a smartphone[21]. Approximately 40-50% of the LMIC population is connected to mobile internet[20 22], with rates up to 60% in China[23]. This

For peer review only

renders mobile health (mHealth) a promising method for health delivery in underserved populations, including the improvement of cardiovascular risk factors[24 25].

We have developed a coach-supported mHealth intervention to reduce dementia risk by addressing common cardiovascular risk factors via lifestyle changes, building on the existing HATICE internet platform. Our aim is to assess the effectiveness and implementation of this smartphone intervention on dementia risk in older people at increased risk of dementia from a low SES population in the UK and from the general population in Beijing, China.

For peer review only

Methods

Study design

PRODEMOS is a multinational, prospective, open-label blinded endpoint (PROBE) trial with 18 months intervention and follow-up. The study follows a hybrid effectiveness-implementation design, taking a dual focus on assessing effectiveness and implementation outcomes[26 27]. The Amsterdam University Medical Centre (Amsterdam UMC) is the coordinating centre.

Study population and recruitment

The study population will consist of community-dwelling older adults aged 55-75 years, of low SES in the UK and of any SES in China, who have ≥2 dementia risk factors and own a smartphone. Low SES in the UK is operationalised as living in a postal code area ranked as equal to or less than the lowest third decile of the index of multiple deprivation[28]. Eligibility criteria are similar for both countries, except for criteria for obesity, based on differences in national prevention guidelines[29] (table 1).

Recruitment will take place in the Eastern Clinical Research Network (National Institutes of Health Research) region of the UK and in the Beijing and Tai'an cities, China. In the UK, recruitment has started in January 2021 and will be undertaken in approximately 10-15 general practices (GPs). A random computer selection of participants living in the designated postal code areas meeting the age criterion and having ≥1 known dementia risk factor according to the GP registry will be approached through an information letter, inviting them to contact the local study centre. In China, participants will be recruited from seven hospitals through advertisements on hospital websites, targeted recruitment via local social media (WeChat), or direct approach by nurses and physicians. In China, recruitment is expected to start mid 2021.

Intervention and control condition

Central to our study is the PRODEMOS platform, which interconnects the assessor portal, participant app, and the coach portal (Figure 1). The assessor portal facilitates blinded collection of baseline and outcome assessments for all participants. The intervention and control condition are both delivered through a smartphone app, which, in the case of intervention participants, allows communication with the coach portal. Data from the assessor portal, participant app, and coach portal can be extracted through a researcher portal and stored in a central database. The PRODEMOS platform was built in close collaboration between software developers and researchers from Amsterdam UMC, University of Cambridge, Brighton and Sussex Medical School, Capital Medical University in Beijing, health coaches, and representatives of the target population from both countries. The internet platform previously used in the HATICE trial served as the basis for the PRODEMOS

For peer review only

platform[30]. In addition to the transition of the participants' end into a mobile app, adjustments were made to the platform in repeated cycles of interaction with end-users. In an iterative process, experiences, needs, and wishes from the target population and health coaches regarding the app and coach-support, gained through interviews and focus groups, served as a guideline for further development.

Participants have only access to one of two versions of the participant app. Participants randomised to the intervention condition will have access to an interactive smartphone application in their own language (English in the UK, Mandarin in China). The intervention app facilitates coach-supported self-management of seven dementia risk factors, including overweight, unhealthy diet, insufficient physical activity, smoking, hypertension, dyslipidaemia, and diabetes. Participants can set personal goals for lifestyle change, following the SMART (specific, measurable, achievable, realistic, time-bound) principle. Participants receive automated reminders to enter measurements (e.g. number of steps, blood pressure) for these goals, facilitating progress monitoring. The intervention participants will receive support from an experienced lifestyle coach, who is trained in motivational interviewing and works according to a coach protocol based on current guidelines for risk factor management. Regular training sessions in each country will enhance uniformity in coaching procedures, taking cultural differences into account. During the baseline visit, after randomisation, the coach discusses the participant's dementia risk profile and a first lifestyle goal will be set through the app. After the baseline visit, all communication between participant and coach will take place through the messaging functionality. Through the coach portal, the coach can view goals and measurements, send tailored education modules, and can offer remote support to facilitate sustainable behaviour change.

Participants randomised to the control condition will have access to the control app, which is similar in appearance, but only contains education material, lacking interactive features and coach-support. During the baseline visit, control participants will receive concise feedback on their risk profile.

The PRODEMOS intervention in its current design is positioned as add-on to existing care.

<u>Primary and secondary outcomes</u>

Following a type-II hybrid design, primary outcomes for effectiveness and implementation are equally important. The primary effectiveness outcome is the change in the Cardiovascular risk factors, Aging and Incidence of Dementia (CAIDE) dementia risk score between baseline and 18-months follow-up[31]. The main secondary effectiveness outcomes include change in the individual modifiable components of the primary outcome, change in ten-year CVD risk, cost-effectiveness, and certain clinical outcomes such as incidence of mild cognitive impairment (MCI) and dementia. The operationalisation of all effectiveness outcomes is listed in table 2.

For peer review only

Implementation outcomes include acceptability, adoption, appropriateness, feasibility, fidelity, coverage, sustainability, and costs of the implementation. User statistics, including data on goals set, messages sent, and education items read, will be analysed to assess adoption and sustained use of the platform. In-depth interviews with participants and coaches will focus on user-experiences, particularly with respect to barriers and facilitators for (sustained) platform use. All implementation outcomes and evaluation methods are shown in table 3.

Study logistics and data collection

The trial design is visualised in figure 2. All participants will receive one phone call and make three visits to a study venue during the study. Data is collected in electronic case report forms (eCRF) that are accessible through the assessor portal (figure 1).

Eligibility criteria that can be assessed remotely will be checked by a local research team member through the screening phone call. During the subsequent screening visit, informed consent (supplement) will be obtained and final eligibility will be assessed by administering the Mini-Mental State Examination (MMSE), measuring blood pressure, BMI, waist circumference, total cholesterol (capillary blood sample in the UK; venous blood sample in China), and assessing physical activity, smoking behaviour, and a brief medical history. Weight will be measured with a calibrated scale, blood pressure will be measured twice with a calibrated, automated blood pressure device. Screening visits will be performed by (GP) nurses and local research team members specifically trained to perform these measurements, and will take place at the GP surgery or a nearby community venue. Standard Operating Procedures will be used to achieve uniform measurements within and between countries.

After the screening visit, all participants will fill in eight self-assessment questionnaires in the PRODEMOS app. These questionnaires will be used to assess secondary outcomes (i.e. physical activity, quality of life, well-being, disability, depressive symptoms, self-management, anxiety, and diet, table 2) and potential barriers for lifestyle behaviour change, which can inform coaches to tailor their coaching strategy. Seven of these questionnaires (i.e. International Physical Activity Questionnaire-Short Form (IPAQ-SF), EuroQol 5 dimensions (EQ-5D), ICEpop CAPability measure for Adults (ICECAP-A), WHO Disability Assessment Schedule (WHODAS) 2.0, Geriatric Depression Scale (GDS), Partners In Health (PIH), and Hospital Anxiety and Depression Scale–Anxiety (HADS-A)) have been externally validated in both Western and Chinese populations[32-45]. Owing to obvious cultural differences, we decided to use two different diet questionnaires that were validated in the UK and Chinese population respectively (short-form food frequency questionnaire and Kadoorie Biobank food frequency questionnaire)[46 47].

The baseline visit will be conducted face-to-face by the health coach at the GP practice or local community venue. During this visit, self-assessment questionnaires are reviewed, relevant medical

For peer review only

history and medication use are recorded, and participants are randomly assigned to one of the treatment conditions. Only intervention participants will set a first lifestyle goal together with the coach, according to their dementia risk profile.

All participants will receive periodic adverse event (AE) questionnaires in the app, assessing incident dementia, MCI, CVD, and diabetes. All self-reported outcomes will be verified with the participant's treating physician.

After 18 months, the questionnaires and all measurements performed during the screening- and baseline visit are repeated during the final visit.

Randomisation and blinding

After completion of the baseline assessments, participants will be individually randomised in a 1:1 ratio, stratified by country, using a central, computer-generated sequence. Participating cohabiting partners will be allocated to the same study condition. Complete blinding of participants is not possible, owing to the nature of the intervention. Participants will be informed that they will be randomised to one of two lifestyle apps, without further details. All outcome assessments will be done by an independent assessor unaware of treatment allocation.

Safety and privacy

Due to the nature of the intervention, serious AE's are unlikely to occur, and we consider the intervention low risk. A data safety and monitoring board is not installed.

Some precautions are taken to optimise participant safety. Firstly, regardless of their study allocation, participants will be referred to their GP or treating physician if deemed necessary based on their baseline or outcome parameters and local guidelines. Secondly, AE's will be monitored through three six-monthly questionnaires, for which participants will receive notifications on their smartphone and reminders through e-mail (UK) or SMS (China). If the participant is not able to fill in the questionnaire, an informant can be contacted. A blinded researcher will, with explicit permission gained through the informed consent procedure, cross-check all reported AE's by consulting the participant's GP or treating physician. Thirdly, the PRODEMOS platform is built in accordance with the highest security requirements in healthcare. It complies with NEN 7510, the Health Insurance Portability and Accountability Act (HIPAA), ISO133485, and General Data Protection Regulation (GDPR).

Protocol adjustments due to COVID-19 pandemic

As a result of the COVID-19 pandemic and related local research restrictions, certain adjustments have been made to the original study protocol as published on ISRCTN. Firstly,

For peer review only

recruitment was planned to start in early 2020, but had to be suspended until January 2021. Second, as it is difficult to predict the development of the pandemic and associated restrictions, we have slightly amended the study protocol to allow for flexible measurement procedures at baseline that can be operationalised in either one or two face-to-face visits, and for a flexible intervention duration of 12-18 months. However, we will strive for a follow-up period of 18 months in as many participants as possible.

Patient and Public Involvement

We have received valuable input into the design of the study and mHealth platform from multiple interactive sessions with GPs, health coaches, researchers, representatives of people living with dementia, community leaders, and policy makers. Needs and views regarding the intervention were assessed through interviews and focus groups with potential end-users in both countries. All patient-facing material used in the UK has been reviewed by potential end-users. Qualitative evaluations of the pilot study with research staff, coaches and patient participants were used to refine the intervention and study procedures.

For peer review only

Statistical analysis

Sample size

The CAIDE score will be used as primary effectiveness outcome. We decided to use a difference of 0.186 points on the CAIDE score between the average of both study groups as a minimum target threshold, because this difference was observed in the Prevention of Dementia by Intensive Vascular care (preDIVA) trial after two years (p=0.005; intervention group -0.290±1.47 SD, control group -0.104±1.36 SD). Attrition after two years of follow-up was 21% in this study[14]. With 80% power, a 0.05 two-sided significance level, accounting for 21% attrition, and a mean difference in change in CAIDE of 0.186, the required sample size is estimated to be 2319 participants. To allow for unexpected factors we raised this to 2400.

Data analysis

The effect on the CAIDE score will be analysed using linear mixed effects models according to the intention-to-treat principle, taking clustering within partner pairs and country into account by testing best fit for random intercept and/or slope. If needed, we will adjust for baseline imbalances and take clustering of the intervention within centre and/or coach into account. No imputation of the CAIDE score will be done for the primary analysis. In sensitivity analyses, we will use multiple imputation to assess the impact of missing items needed to calculate the CAIDE score, provided there are no indications that the variables are missing not at random (MNAR), and a per protocol analysis for those adherent to the intervention will be performed. Moreover, we will explore the interaction of intervention duration with the effect of the invention by adding an interaction term (intervention duration*randomisation group) to the main model. This will give insight into the potential additional intervention effect in participants with a follow-up time of less than 18 months.

Subgroup analyses will be performed for country, sex, age-group, history of CVD yes/no, number of risk factors, willingness to change lifestyle (assessed with one question during the baseline visit), participation with(out) a participating partner, having the same coach during the full length of the study, and the number of goals set. For all these factors, interaction terms will be included to test for between-subgroup differences in intervention effects.

The effect on individual modifiable components of the CAIDE score and 10-year CVD risk scores will be analysed using linear mixed effects models according to the intention to treat principle, taking clustering within partner pairs and country into account. Self-assessment scales, which are mostly ordinal, will be regarded as linear scales if there are at least four categories and the 'distance' between the categories can be regarded equal. Poisson regression or zero-inflated models may be applied to

For peer review only

distributions resembling count or zero-inflated data. The choice of the final model will be a compromise between optimal fit and interpretability of the results for a general clinical public.

Prevalence ratios will be used for self-assessment instruments with defined cut-offs for the presence or absence of a condition, e.g. ‘depressive symptoms’ for a GDS>5. For (clinical) dichotomous outcomes, such as incident CVD, dementia or mortality, Cox-proportional hazard models will be used with time since baseline as timescale. A sensitivity analysis will be performed using age as timescale.

The full analysis plan, including the health economic analysis plan entailing the cost-consequence analysis of the within trial results, the cost-effectiveness analyses, and the cost-utility analysis, and hypotheses for the subgroup analyses, is published on the ISRCTN website: http://www.isrctn.com/ISRCTN15986016.

For peer review only

Pilot study

Between December 2019 and March 2020, a six-week pilot study was conducted in the Brighton and Sussex area, UK. Since the main aim was to test study logistics and functionality of the intervention app, participants were randomised in a 3:1 (intervention:control) ratio. An invitation letter was sent to 600 potentially eligible patients from two GPs. The response rate was 14.8% (n=89), of whom 21 participants (3.5%) could be included. Main reasons for exclusion were not living in the designated postal code area and having <2 dementia risk factors. Participants had a median age of 69 years and 12 (57%) were male. Fifteen participants were allocated to the intervention group and six to the control group.

During the pilot study, 10 of 15 intervention participants set at least one goal (range 1–8 goals). Goals were set in five domains, including physical activity, healthy diet, body weight, blood pressure, and cholesterol. Six of ten participants entered goal-related measurements (range 2–243 measurements). All intervention participants used the chat functionality to consult the coach. In total 278 messages were sent back and forth, i.e. on average 3 messages per intervention participant per week.

The pilot study was evaluated through qualitative sessions with participants and coaches. The main adjustments based on the participants' feedback included improvements to the chat functionality (allowing attachments, larger font-size), simplification of the functionality to enter and view measurements, setting the first goal together with the coach, and more detailed instructions for app use through an instruction video and written manual. Based on feedback from the coaches, we improved the functionalities for population management in the coach portal, including an input screen to make notes about individual participants and a functionality to send education material to (groups of) participants.

A similar pilot study will be conducted in China, to test platform functionality and study logistics in all seven participating trial centres.

For peer review only

Discussion

In the PRODEMOS study we will investigate the implementation of a self-management mHealth intervention with remote coaching and its effect on dementia risk over 18 months. We will target people aged 55-75 years with elevated dementia risk of low SES in the UK and of any SES in the Beijing and Tai'an cities in China, as these populations are usually not reached by preventive strategies and may benefit most. User data and qualitative analysis of our pilot study suggest that our mHealth application, after further adaptations to improve attractiveness and usability, is now ready to be studied in older adults who are interested in participating in a study on lifestyle change to lower their overall dementia risk.

The HATICE trial has shown that a coach-supported internet platform can improve cardiovascular risk factors in European elderly. Although we build on these experiences, the modality (i.e. app instead of internet platform) and target population are different. The resulting uncertainty that there would be a similar benefit of our intervention renders the use of a hybrid effectiveness-implementation design highly suitable[27].

<u>Strengths</u>

Chronic disease risk is largely affected by socioeconomic factors, including psychological, cultural, and economic characteristics, requiring preventive strategies that take these aspects into account[48]. In PRODEMOS we aim to support individuals by offering intensive human support through the app, and by aligning the intervention with the healthcare system. In order to eventually embed a complex prevention intervention into primary healthcare, it is crucial to involve and consult all stakeholders, such as GPs, practice nurses, and end-users[49]. In the current hybrid effectiveness-implementation study we take some first steps to explore the possibilities and challenges for embedding the intervention in existing healthcare. This study will provide concrete evidence of the scale of the change that might be achieved for individuals at risk, whether and how this approach is taken up within diverse populations.

The PRODEMOS study is designed as one trial, recruiting participants in two different countries, increasing the external validity of the results. Overall, both countries will follow the same research protocol and highly similar standard operating procedures and will investigate similar interventions. Through semi-structured interviews among elderly in Beijing and the UK, we learned that needs and wishes regarding lifestyle behaviour change through mHealth are largely similar (manuscripts currently being drafted). Therefore, the Chinese and UK intervention will share the same functionalities and coaching procedures. Given obvious cultural and healthcare-related differences, certain aspects of the study logistics, lifestyle support, and lay-out of the app had to be culturally

For peer review only

adjusted. In a pre-planned subgroup analysis, we will assess both effectiveness and implementation outcomes for both countries separately.

Limitations

The study may yield some limitations. Firstly, the optimal age range for trials on dementia risk reduction is unknown[15]. There is a trade-off between potentially more effective treatments in midlife and the chance to detect treatment effects on cognitive outcomes in late-life[4]. As in the current study we are assessing both a dementia risk score and clinical outcomes, we have taken a pragmatic approach, targeting individuals aged 55-75 years[15].

Secondly, change in CAIDE dementia risk score is not easily translated into incidence of dementia. However, although not specifically designed as RCT outcome measure, the CAIDE score can detect change over time[50].

A third potential limitation is that, owing to the nature of the intervention, blinding of the participants is only partly possible. A certain degree of contamination might occur, especially in communities that live closely together. The study logistics and intervention are designed in such a way as to limit contact between participants after randomization.

Finally, the results of the baseline measurements will be revealed to all participants, potentially leading to treatment effects in both study conditions. Also, behaviour of participants and their treating physicians may change in both study conditions as a reaction to the awareness of being part of the study (Hawthorne-effect). Both mechanisms will perhaps mask (part of) the "true" contrast in dementia risk between the intervention and control condition.

For the planned health economic analyses we will rely on economic modelling, based on the intermediate outcomes reflecting risk of dementia and CVD and assumptions on their causality with clinical endpoints dementia and CVD, because the study is not designed nor powered to detect an effect on these clinical endpoints.

The high prevalence of dementia, lower provision of high-quality cardiovascular preventive care in LMIC and lower uptake of such programmes in western low SES populations require affordable and straightforward preventive strategies. If proven effective and implementable, our pragmatic smartphone intervention facilitates widespread use and reduction of dementia risk for hard-to-reach populations across the globe.

For peer review only

Table 1. Overview of in- and exclusion criteria.

Inclusion criteria

- Age ≥ 55 years ≤ 75 years
- Living in a postal code area ranked as equal to or less than the lowest 3rd decile of IMD[a]
- Good proficiency of the national language (English in UK, Mandarin in China)
- Possession of a smartphone
- ≥ 2 Dementia risk factors:
  - Insufficient physical activity (self-reported intermediate or vigorous activity of < 150 minutes per week)
  - Active smoking (self-reported use of any sort of tobacco in any quantity)
  - Depression:
    - Current diagnosis by specialist or GP or;
    - History of treatment for depression (i.e. drug therapy or psychotherapy)
  - Manifest cardiovascular disease, as diagnosed by specialist or GP
  - Diabetes mellitus:
    - Diagnosed by specialist or GP or;
    - Use of insulin or other blood glucose-lowering medication
  - Hypertension:
    - Diagnosed by specialist or GP or;
    - Use of blood pressure-lowering medication or;
    - Mean of baseline blood pressure measurements of ≥ 140 (systolic) or ≥ 90 (diastolic)
  - Obesity:
    - BMI ≥ 30 (UK), ≥ 28 (China) or;
    - Baseline waist circumference ≥ 102 cm (men in UK), 90 cm (men in China), 88 cm (women in UK), 85 cm (women in China)
  - Dyslipidemia:
    - Diagnosed by specialist or GP or;
    - Use of lipid-lowering medication or;
    - Baseline total cholesterol ≥ 5.0 mmol/L[a]

Exclusion criteria

- Manifest dementia, as diagnosed by specialist or GP
- MMSE < 24 (participants with ISCED level of > 1), MMSE <21 (participants with ISCED level of 1)
- Any condition expected to limit 18-months follow-up, including metastasized malignancy or other terminal illnesses
- Smartphone illiteracy, defined as not being able to send a message from a smartphone
- Visual impairment interfering with operation of a smartphone
- Participating in another RCT on behaviour change
- Present severe alcohol or illicit drug abuse

[a] Applies only to participants in United Kingdom. BMI = Body Mass Index; GP = general practitioner; IMD = Index of Multiple Deprivation; ISCED = International Standard Classification of Education; MMSE = Mini Mental State examination; RCT = Randomised Controlled Trial; UK = U=1 ofnited Kingdom.

For peer review only

Table 2. Effectiveness outcomes.

**Primary outcome**		
CAIDE score (range 0-15), which is comprised of and calculated from:		Points
Age	<47 years	0
	47-53	3
	>53	4
Education	≥10 years	0
	7-9 years	2
	<7 years	3
Gender	Female	0
	Male	1
Systolic blood pressure	≤140 mmHg	0
	>140 mmHg	2
BMI	≤30 kg/m^2	0
	>30 kg/m^2	2
Total cholesterol	≤6.5 mmol/L	0
	>6.5 mmol/L	2
Physical activity [a]	Yes	0
	No	1

**Secondary outcomes**	
Individual modifiable components of the CAIDE score[b]	Estimated 10-year cardiovascular risk
Number of uncontrolled risk factors	LIBRA dementia risk score
Active smoking	Number of hospital admissions
Medication adherence	Diet [e]
Number of drugs	Disability [f]
Incident dementia[c]	Anxiety [g]
Incident MCI[c]	Self-management [h]
Incident cardiovascular disease[c,d]	Depressive symptoms [i]
Incident diabetes[c]	Quality of life [j]
All-cause mortality	Cost-effectiveness

[a] assessed according to WHO standard for physical activity of at least 150 minutes per week; [b] physical activity assessed with the International Physical Activity Questionnaire Short Form; [c] self-reported and cross-checked with GP file; [d] Defined as myocardial infarction or stroke; [e] assessed with short-form food frequency questionnaire (UK) and China Kadoorie Biobank food frequency questionnaire (China); [f] assessed with the WHO Disability Assessment Schedule; [g] assessed with the Hospital Anxiety and Depression Scale – Anxiety; [h] assessed with the Partners In Health; [i] assessed with the Geriatric Depression Scale; [j] assessed with the ICEpop CAPability measure for Adults and EuroQol 5 dimensions 3 levels. BMI = Body Mass Index; CAIDE = Cardiovascular Risk Factors, Aging and Dementia; LIBRA = Lifestyle for Brain Health; MCI = Mild Cognitive Impairment; UK = United Kingdom.

For peer review only

501 **Table 3. Summary of implementation research methods and outcomes.**

Method	Outcome	Measurement	Population[a]	Timing of assessment
Quantitative	Coverage	(Non)response rates, comparison characteristics of participants with eligible population	Potential target population	At baseline
	Adoption	Utilisation, usage, and uptake	Intervention participants, coaches	After two weeks
	Appropriateness	Short questionnaire of perceived fit or relevance in the target population and the coaches	Intervention participants, coaches	After 3 months and at study end
	Acceptability	Short questionnaire of agreeability, user-friendliness, credibility	Intervention participants, coaches	After 3 months and at study end
	Sustainability	Adherence, dropout	Intervention participants, dropouts[b]	Throughout the study
	Cost	Implementation costs	N.a.	N.a.
Qualitative	Feasibility	The extent to which the mHealth intervention can be carried out → practical and social barriers/facilitators	Intervention participants, dropouts[b], coaches	After 3 months and at study end
	Appropriateness	Perceived fit or relevance in the target population	Intervention participants, dropouts[b], coaches	After 3 months and at study end
	Acceptability	Agreeability, user-friendliness, credibility	Intervention participants, dropouts[b], coaches	After 3 months and at study end
	Fidelity	Degree to which the mHealth application is implemented compared to the original protocol	N.a.	After the study

502 [a] For all analyses a Chinese and UK population will be involved. [b] Study dropouts will be asked to participate in a short exit-
503 interview. mHealth = mobile health. AE = adverse event.
504
505
506
507
508
509
510
511
512
513
514

Figure 1. Overview of the PRODEMOS platform and its functionalities

Legend: Main features of coach portal: viewing and adjusting details of goals and measurements; sending and receiving chat messages to and from participants; sending education- and news items. Main features of intervention app: setting and adjusting goals; entering measurements; sending and receiving chat messages to and from coach; reading education- and news items automatically pushed by platform or received from coach; receiving periodic adverse event questionnaires and self-assessment questionnaires. Main features of assessor portal: blinded collection of participant data through electronic CRFs and questionnaires. The control application has similar connections with the assessor portal and the researcher portal / database, but is not connected to the coach portal.

Figure 2. Trial design

Legend: AE = Adverse Event, UK = United Kingdom.

For peer review only

Ethics and dissemination

The PRODEMOS trial is sponsored in the UK by the University of Cambridge and is granted ethical approval by the London–Brighton&Sussex Research Ethics Committee (REC Reference:20/LO/01440). In China, the trial is approved by the Medical Ethics Committees of Capital Medical University (CMU), Beijing Tiantan Hospital, Beijing Geriatric Hospital, Chinese People's Liberation Army General Hospital, Taishan Medical University, and Xuanwu Hospital. Data will be exported in a pseudonymised format according to prevailing guidelines on good clinical practice in both countries. Only anonymised data will be exchanged between the UK, China, and the Netherlands. The exported data will be stored centrally on a protected server in the Netherlands, which is compatible with the highest standards of data management in medical research. Results will be published in a peer-reviewed journal.

Author affiliations

[1] Department of General Practice, Amsterdam University Medical Centre, University of Amsterdam, Amsterdam, The Netherlands
[2]Department of Neurology, Amsterdam University Medical Centre, University of Amsterdam, Amsterdam, The Netherlands
[3]Beijing Key Laboratory of Clinical Epidemiology, School of Public Health, Capital Medical University, Beijing, China
[4]School of Medical and Health Sciences, Edith Cowan University, Perth, Australia
[5]INSERM/University of Toulouse UMR1027, Toulouse, France
[6]Department of Epidemiology and Public Health, Toulouse University Hospital, Toulouse, France
[7]Cambridge Public Health, University of Cambridge, Cambridge, United Kingdom
[8]Alzheimer Europe, Luxemburg City, Luxemburg
[9]Department of Primary Care and Public Health, Brighton and Sussex Medical School, Brighton, United Kingdom
[10]Vital Health Software, Ede, Netherlands
[11]Department of Public and Occupational Health, Amsterdam University Medical Centre, University of Amsterdam, Amsterdam, The Netherlands
[12]Alzheimer Centre Limburg, School for Mental Health and Neuroscience, Maastricht University Medical Centre+, Maastricht, the Netherlands
[13]Division of Neurogeriatrics, Department of Neurobiology, Care Sciences and Society, Karolinska Institutet, Stockholm, Sweden
[14]School of Public Health, Shandong First Medical University & Shandong Academy of Medical Science, Tai'an, Shandong, China
[15]Beijing Neurosurgical Institute, Beijing, China
[16]Centre for Cognitive Disorders, Beijing Geriatric Hospital, Beijing, China
[17] Department of Geriatrics, The Second Medical Centre & National Clinical Research Centre for Geriatric Diseases, Chinese PLA General Hospital, Beijing, China
[18]Fuzhou Comvee Network & Technology Co., Ltd, Fuzhou, China
[19]Department of Neurology, Xuanwu Hospital, Capital Medical University, Beijing, China
[20]Health Management Institute, The Second Medical Centre & National Clinical Research Centre for Geriatric Diseases, Chinese PLA General Hospital, Beijing, China
[21]Centre for Cognitive Neurology, Department of Neurology, Beijing Tiantan Hospital, Capital Medical University, Beijing, China
[22]Department of Neurology, Donders Institute for Brain, Behaviour and Cognition, Radboud University Medical Centre, Nijmegen, The Netherlands

Acknowledgements

The authors thank the European Union's Horizon 2020 research and innovation programme and National Key R & D Programme of China for the funding of the trial. The authors thank Lonneke van Vught for her substantial contribution in the early phase of the trial design. The authors thank Kevin Hekert, Louwrens Knulst, and Michiel van Dam for their substantial contribution in building the platform. The authors thank Jinxia Zhang, Xiaoyu Zhang, Mingyang Cao, Bin Jiang, Siqi Ge, Maolong Gao, Mo Li, Nayan Huang, Danning Li, Mingyue He, Weijiao Zhang, Huiying Guan, Jinghui Li, Yan Gong, Na Niu, Xiang Jia, Fei Wang, Jing Sun, Liyong Wu, Dan Li, Baoliang Sun, Hui Yuan, Guohua Wang, Xizhu Xu, Cancan Li, Wenran Zhang, Juan Du, and Libin Song for their contributions to the trial logistics. The authors thank all patient participants and other stakeholders who took part in PPI groups for their contribution to the platform and study design.

Author contributions

EE was responsible for the drafting of the manuscript. ER, EMvC, WAvG, MHB, CB, and WW were responsible for the study conception. ER, EMvC, MHB, MS, SA, CB (Cindy Birck), NC, EF, JG, WAvG, HvM, Wenzhi Wang, YW, AW, WW (Wei Wang), and CB (Carol Brayne) were responsible for the design of the trial. EE, MH, LB, RB, AG, RH, HH, DL, HL, JL, MM, YN, SS, XY, YY, QZ, and WZ were involved in trial design and coordination. All other authors were responsible for critically revising the manuscript. All authors approved the final version of the manuscript.

Funding

This project has received funding from the European Union's Horizon 2020 research and innovation programme under grant agreement No 779238 and the National Key R&D Programme of China (2017YFE0118800).

For peer review only

Competing interests statement

We have no competing interests to declare.

Word count

4000

Ethics approval

The PRODEMOS trial is sponsored in the UK by the University of Cambridge and is granted ethical approval by the London – Brighton & Sussex Research Ethics Committee (REC Reference: 20/LO/01440). In China the trial is approved by the Medical Ethics Committees of Capital Medical University (CMU), Beijing Tiantan Hospital affiliated to CMU, Beijing Geriatric Hospital, the Chinese People's Liberation Army General Hospital, Taishan Medical University, and the Xuanwu Hospital affiliated to CMU.

For peer review only

Literature

1. Prince M. World Alzheimer Report 2015. The global impact of dementia. An analysis of prevalence, incidence, cost and trends. 2015
2. Yaffe K, Falvey C, Harris TB, et al. Effect of socioeconomic disparities on incidence of dementia among biracial older adults: prospective study. *BMJ* 2013;347:f7051. doi: 10.1136/bmj.f7051 [published Online First: 2013/12/21]
3. Livingston G, Huntley J, Sommerlad A, et al. Dementia prevention, intervention, and care: 2020 report of the Lancet Commission. *Lancet* 2020;396(10248):413-46. doi: 10.1016/S0140-6736(20)30367-6 [published Online First: 2020/08/03]
4. Qiu C, Winblad B, Fratiglioni L. The age-dependent relation of blood pressure to cognitive function and dementia. *Lancet Neurol* 2005;4(8):487-99. doi: 10.1016/S1474-4422(05)70141-1
5. Qiu C, Xu W, Fratiglioni L. Vascular and psychosocial factors in Alzheimer's disease: epidemiological evidence toward intervention. *J Alzheimers Dis* 2010;20(3):689-97. doi: 10.3233/JAD-2010-091663 [published Online First: 2010/02/26]
6. Barnes DE, Yaffe K. The projected effect of risk factor reduction on Alzheimer's disease prevalence. *Lancet Neurol* 2011;10(9):819-28. doi: 10.1016/S1474-4422(11)70072-2 [published Online First: 2011/07/22]
7. Kivipelto M, Mangialasche F, Ngandu T. Lifestyle interventions to prevent cognitive impairment, dementia and Alzheimer disease. *Nat Rev Neurol* 2018;14(11):653-66. doi: 10.1038/s41582-018-0070-3 [published Online First: 2018/10/07]
8. Andrieu S, Coley N, Lovestone S, et al. Prevention of sporadic Alzheimer's disease: lessons learned from clinical trials and future directions. *Lancet Neurol* 2015;14(9):926-44. doi: 10.1016/S1474-4422(15)00153-2 [published Online First: 2015/07/28]
9. van Middelaar T, van Vught LA, van Gool WA, et al. Blood pressure-lowering interventions to prevent dementia: a systematic review and meta-analysis. *J Hypertens* 2018;36(9):1780-87. doi: 10.1097/HJH.0000000000001829 [published Online First: 2018/06/22]
10. Sprint Mind Investigators for the SPRINT Research Group. Effect of Intensive vs Standard Blood Pressure Control on Probable Dementia: A Randomized Clinical Trial. *JAMA* 2019;321(6):553-61. doi: 10.1001/jama.2018.21442 [published Online First: 2019/01/29]
11. Hughes D, Judge C, Murphy R, et al. Association of Blood Pressure Lowering With Incident Dementia or Cognitive Impairment: A Systematic Review and Meta-analysis. *JAMA* 2020;323(19):1934-44. doi: 10.1001/jama.2020.4249 [published Online First: 2020/05/20]
12. Luchsinger JA, Reitz C, Honig LS, et al. Aggregation of vascular risk factors and risk of incident Alzheimer disease. *Neurology* 2005;65(4):545-51. doi: 10.1212/01.wnl.0000172914.08967.dc [published Online First: 2005/08/24]
13. Yaffe K. Metabolic syndrome and cognitive disorders: is the sum greater than its parts? *Alzheimer Dis Assoc Disord* 2007;21(2):167-71. doi: 10.1097/WAD.0b013e318065bfd6 [published Online First: 2007/06/05]
14. Moll van Charante EP, Richard E, Eurelings LS, et al. Effectiveness of a 6-year multidomain vascular care intervention to prevent dementia (preDIVA): a cluster-randomised controlled trial. *Lancet* 2016;388(10046):797-805. doi: 10.1016/S0140-6736(16)30950-3 [published Online First: 2016/07/31]
15. Richard E, Andrieu S, Solomon A, et al. Methodological challenges in designing dementia prevention trials - the European Dementia Prevention Initiative (EDPI). *J Neurol Sci* 2012;322(1-2):64-70. doi: 10.1016/j.jns.2012.06.012 [published Online First: 2012/07/24]
16. Coley N, Andrieu S, Gardette V, et al. Dementia prevention: methodological explanations for inconsistent results. *Epidemiol Rev* 2008;30:35-66. doi: 10.1093/epirev/mxn010 [published Online First: 2008/09/10]

For peer review only

17. Eggink E, Moll van Charante EP, van Gool WA, et al. A Population Perspective on Prevention of Dementia. *J Clin Med* 2019;8(6) doi: 10.3390/jcm8060834 [published Online First: 2019/06/20]
18. Beishuizen CR, Stephan BC, van Gool WA, et al. Web-Based Interventions Targeting Cardiovascular Risk Factors in Middle-Aged and Older People: A Systematic Review and Meta-Analysis. *J Med Internet Res* 2016;18(3):e55. doi: 10.2196/jmir.5218 [published Online First: 2016/03/13]
19. Richard E, Moll van Charante E, Hoevenaar-Blom MP, et al. Healthy ageing through internet counselling in the elderly (HATICE): a multinational, randomised controlled trial. 2019;1(8):e424-e34. doi: https://doi.org/10.1016/S2589-7500(19)30153-0
20. Bahia K, Suardi S. Connected society, the state of mobile internet connectivity in 2019, 2019.
21. Statista. Do you personally use a smartphone?* - by socio-economic group 2019 [Available from: https://www.statista.com/statistics/300421/smartphone-usage-in-the-uk-by-socio-economic-group/ accessed 28-01-2021.
22. O'Dea S. Smartphone penetration rate by country 2018

2018 [Available from: https://www.statista.com/statistics/539395/smartphone-penetration-worldwide-by-country/ accessed 28-01-2021.
23. Smartphone penetration rate in China from 2015 to 2023 [Available from: https://www.statista.com/statistics/321482/smartphone-user-penetration-in-china/ accessed 28-01-2021.
24. Coorey GM, Neubeck L, Mulley J, et al. Effectiveness, acceptability and usefulness of mobile applications for cardiovascular disease self-management: Systematic review with meta-synthesis of quantitative and qualitative data. *Eur J Prev Cardiol* 2018;25(5):505-21. doi: 10.1177/2047487317750913 [published Online First: 2018/01/10]
25. Piette JD, List J, Rana GK, et al. Mobile Health Devices as Tools for Worldwide Cardiovascular Risk Reduction and Disease Management. *Circulation* 2015;132(21):2012-27. doi: 10.1161/CIRCULATIONAHA.114.008723 [published Online First: 2015/11/26]
26. Curran GM, Bauer M, Mittman B, et al. Effectiveness-implementation hybrid designs: combining elements of clinical effectiveness and implementation research to enhance public health impact. *Med Care* 2012;50(3):217-26. doi: 10.1097/MLR.0b013e3182408812 [published Online First: 2012/02/09]
27. Landes SJ, McBain SA, Curran GM. An introduction to effectiveness-implementation hybrid designs. *Psychiatry Res* 2019;280:112513. doi: 10.1016/j.psychres.2019.112513 [published Online First: 2019/08/23]
28. Payne RA, Abel GA. UK indices of multiple deprivation – a way to make comparisons across constituent countries easier. *Health Statistics Quarterly* 2012;53
29. Wang H, Zhai F. Programme and policy options for preventing obesity in China. *Obes Rev* 2013;14 Suppl 2:134-40. doi: 10.1111/obr.12106 [published Online First: 2013/10/10]
30. Jongstra S, Beishuizen C, Andrieu S, et al. Development and Validation of an Interactive Internet Platform for Older People: The Healthy Ageing Through Internet Counselling in the Elderly Study. *Telemed J E Health* 2017;23(2):96-104. doi: 10.1089/tmj.2016.0066 [published Online First: 2016/07/28]
31. Kivipelto M, Ngandu T, Laatikainen T, et al. Risk score for the prediction of dementia risk in 20 years among middle aged people: a longitudinal, population-based study. *Lancet Neurol* 2006;5(9):735-41. doi: 10.1016/S1474-4422(06)70537-3 [published Online First: 2006/08/18]
32. Wang HM, Patrick DL, Edwards TC, et al. Validation of the EQ-5D in a general population sample in urban China. *Qual Life Res* 2012;21(1):155-60. doi: 10.1007/s11136-011-9915-6 [published Online First: 2011/04/21]
33. Deng HB, Macfarlane DJ, Thomas GN, et al. Reliability and validity of the IPAQ-Chinese: the Guangzhou Biobank Cohort study. *Med Sci Sports Exerc* 2008;40(2):303-7. doi: 10.1249/mss.0b013e31815b0db5 [published Online First: 2008/01/19]

For peer review only

34. Tang C, Xiong Y, Wu H, et al. Adaptation and assessments of the Chinese version of the ICECAP-A measurement. *Health Qual Life Outcomes* 2018;16(1):45. doi: 10.1186/s12955-018-0865-3 [published Online First: 2018/03/14]

35. Üstün T, Kostanjsek N, Chatterji S, et al. Measuring Health and Disability Manual for WHO disability assessment schedule WHODAS 2.0: World Health Organization, 2010.

36. Xie Z, Lv X, Hu Y, et al. Development and validation of the geriatric depression inventory in Chinese culture. *Int Psychogeriatr* 2015;27(9):1505-11. doi: 10.1017/S1041610215000162 [published Online First: 2015/02/24]

37. Xiaofei Z, Hui F, Lawn S, et al. Translation and initial psychometric evaluation of the Chinese version of the partners in health scale. *Biomedical research* 2017;28(16):7322-29.

38. Yang Y, Ding R, Hu D, et al. Reliability and validity of a Chinese version of the HADS for screening depression and anxiety in psycho-cardiological outpatients. *Compr Psychiatry* 2014;55(1):215-20. doi: 10.1016/j.comppsych.2013.08.012 [published Online First: 2013/11/10]

39. EuroQol G. EuroQol--a new facility for the measurement of health-related quality of life. *Health Policy* 1990;16(3):199-208. doi: 10.1016/0168-8510(90)90421-9 [published Online First: 1990/11/05]

40. Yesavage JA, Brink TL, Rose TL, et al. Development and validation of a geriatric depression screening scale: a preliminary report. *J Psychiatr Res* 1982;17(1):37-49. doi: 10.1016/0022-3956(82)90033-4 [published Online First: 1982/01/01]

41. Al-Janabi H, Flynn TN, Coast J. Development of a self-report measure of capability wellbeing for adults: the ICECAP-A. *Qual Life Res* 2012;21(1):167-76. doi: 10.1007/s11136-011-9927-2 [published Online First: 2011/05/21]

42. Al-Janabi H, Peters TJ, Brazier J, et al. An investigation of the construct validity of the ICECAP-A capability measure. *Qual Life Res* 2013;22(7):1831-40. doi: 10.1007/s11136-012-0293-5 [published Online First: 2012/10/23]

43. Reece M, Collins J, Markwick M, et al. The Partners in Health scale: The development and psychometric properties of a generic assessment scale for chronic condition self-management. *Australian Journal of Primary Health* 2003;9:41-52.

44. Craig CL, Marshall AL, Sjostrom M, et al. International physical activity questionnaire: 12-country reliability and validity. *Med Sci Sports Exerc* 2003;35(8):1381-95. doi: 10.1249/01.MSS.0000078924.61453.FB [published Online First: 2003/08/06]

45. Bjelland I, Dahl AA, Haug TT, et al. The validity of the Hospital Anxiety and Depression Scale. An updated literature review. *J Psychosom Res* 2002;52(2):69-77. doi: 10.1016/s0022-3999(01)00296-3 [published Online First: 2002/02/08]

46. Cleghorn CL, Harrison RA, Ransley JK, et al. Can a dietary quality score derived from a short-form FFQ assess dietary quality in UK adult population surveys? *Public Health Nutr* 2016;19(16):2915-23. doi: 10.1017/S1368980016001099 [published Online First: 2016/05/18]

47. Zhu N, Yu C, Guo Y, et al. Adherence to a healthy lifestyle and all-cause and cause-specific mortality in Chinese adults: a 10-year prospective study of 0.5 million people. *Int J Behav Nutr Phys Act* 2019;16(1):98. doi: 10.1186/s12966-019-0860-z [published Online First: 2019/11/07]

48. Havranek EP, Mujahid MS, Barr DA, et al. Social Determinants of Risk and Outcomes for Cardiovascular Disease: A Scientific Statement From the American Heart Association. *Circulation* 2015;132(9):873-98. doi: 10.1161/CIR.0000000000000228 [published Online First: 2015/08/05]

49. Volker N, Williams LT, Davey RC, et al. Implementation of cardiovascular disease prevention in primary health care: enhancing understanding using normalisation process theory. *BMC Fam Pract* 2017;18(1):28. doi: 10.1186/s12875-017-0580-x [published Online First: 2017/02/27]

50. Coley N, Hoevenaar-Blom MP, van Dalen JW, et al. Dementia risk scores as surrogate outcomes for lifestyle-based multidomain prevention trials-rationale, preliminary evidence and

For peer review only

challenges. *Alzheimers Dement* 2020 doi: 10.1002/alz.12169 [published Online First: 2020/08/18]

For peer review only

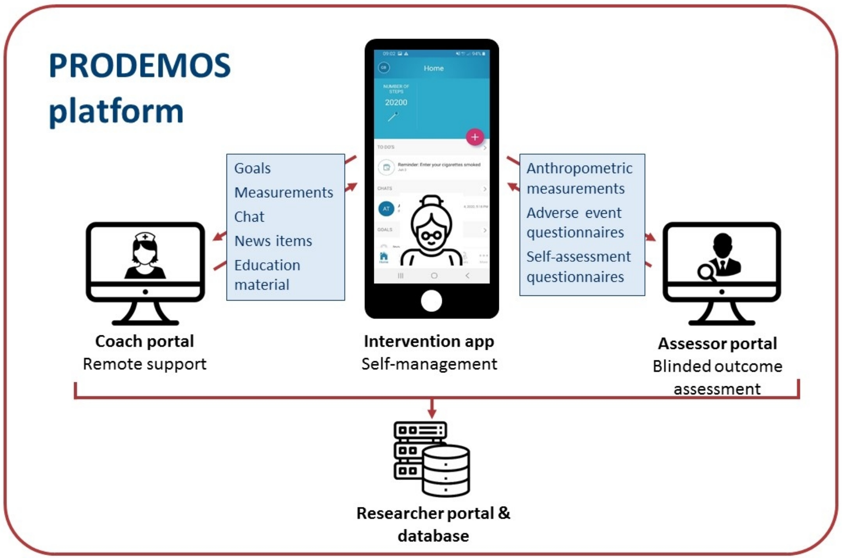

Caption: Figure 1. Overview of the PRODEMOS platform and its functionalities
Legend: Main features of coach portal: viewing and adjusting details of goals and measurements; sending and receiving chat messages to and from participants; sending education- and news items. Main features of intervention app: setting and adjusting goals; entering measurements; sending and receiving chat messages to and from coach; reading education- and news items automatically pushed by platform or received from coach; receiving periodic adverse event questionnaires and self-assessment questionnaires. Main features of assessor portal: blinded collection of participant data through electronic CRFs and questionnaires. The control application has similar connections with the assessor portal and the researcher portal / database, but is not connected to the coach portal.

338x190mm (96 x 96 DPI)

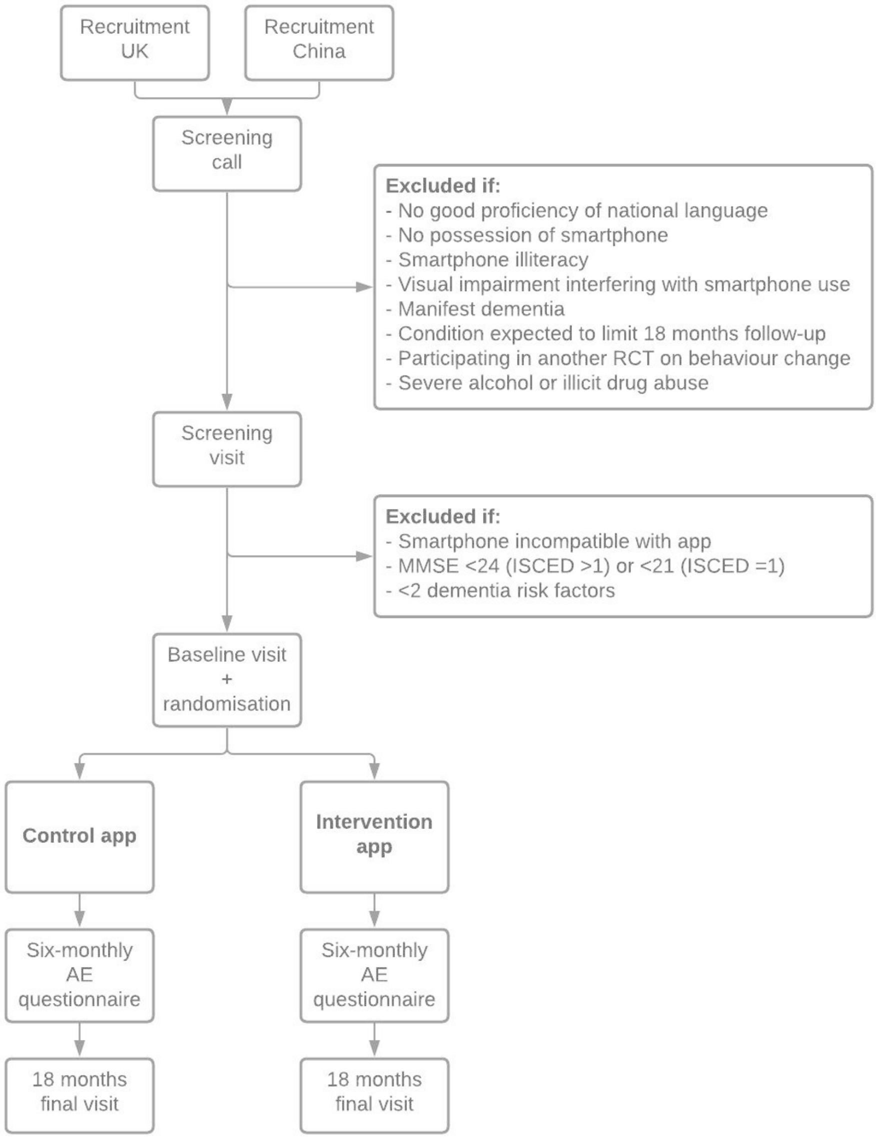

Caption: Figure 2. Trial design
Legend: AE = Adverse Event, ISCED = International Standard Classification of Education, MMSE = Mini-Mental State Examination, RCT = randomized controlled trial, UK = United Kingdom.

161x206mm (160 x 160 DPI)

PRODEMOS

UNIVERSITY OF CAMBRIDGE

Prevention of Dementia using Mobile phone Applications (PRODEMOS): A randomised implementation trial

PARTICIPANT CONSENT FORM

Identification number: ________________________

Prevention Of Dementia Using Mobile Phone Applications (PRODEMOS)

SAMPLE

For peer review only

	Please initial
I confirm that I have read and understood the information sheet (Participant Information Sheet Version 1.1 dated 19/10/2020) for the study: **Prevention of Dementia using Mobile Phone applications**	☐
I have had enough time to ask questions. My questions were answered clearly. I have had enough time to consider participating in the study.	☐
I am aware that participation is voluntary. I am aware that I can withdraw at any stage. I do not need to give a reason.	☐
I understand that my medical care and legal rights will not be affected in any way.	☐
I understand why my data is being collected, and I give permission to use these data for the study purposes as mentioned in the information letter.	☐
I agree to giving a blood sample (fingerprick) for cholesterol testing.	☐
I am aware that I am able to pause the intervention, should my life circumstances change significantly.	☐
I am happy for my GP to know that I am participating in the PRODEMOS study, and for any examination results to be shared with my GP.	☐
I am happy that my data will be stored securely, and I give permission for my data to be stored and managed as mentioned in the participant information sheet.	☐
I am aware that I can ask for my data to be destroyed, prior to 31st December 2022.	☐
I do / do not* give permission to contact me about future research projects.	☐
I am willing to participate in this study.	☐

This project has received funding from the European Union's Horizon 2020 research and innovation programme under grant agreement No 779238

For peer review only - http://bmjopen.bmj.com/site/about/guidelines.xhtml

I would like to receive a copy of the results of this study.<br>(e-mail or postal address):	☐

Name of participant:

Signature of participant:

Date: __ / __ / ____

* Please delete as appropriate

Hereby I declare that I have fully informed the participant about the current research, encouraged the participant to ask questions, and provided adequate time to answer them. In the case of new information that could influence the permission of the participant, I will inform the participant in a timely fashion. I further ensure that 2 copies of the consent form have been completed: 1 copy to be left with the participant, and 1 copy to be filed with the researcher.

Name of researcher:

Signature: Date: __ / __ / ____

2 consent forms to be signed by both parties: 1 copy for the participant; 1 copy for the researcher site file.

SAMPLE

This project has received funding from the European Union's Horizon 2020 research and innovation programme under grant agreement No 779238
For peer review only - http://bmjopen.bmj.com/site/about/guidelines.xhtml

PRODEMOS

UNIVERSITY OF CAMBRIDGE

Prevention of Dementia using Mobile phone Applications

Medical Records Consent

Identification number: |__|-|__|__|__|-|__|__|__|__|

Please Initial

I confirm that I have read and understood the medical records information sheet version 05 dated 04/08/2020 for the PRODEMOS Project and have had the opportunity to ask questions.	☐
I give my permission to PRODEMOS to share my screening, baseline and final outcome visit results with my GP for inclusion in my NHS health record.	☐
I agree to the research staff on the PRODEMOS project accessing my GP medical records.	☐
I agree to PRODEMOS informing my GP of any abnormal test results taken at the screening, baseline, or final visits with the PRODEMOS study.	☐
I understand that my participation will not affect my medical care or legal rights in any way.	☐

SAMPLE

Name of Participant:

Signature of Participant

Date: ___ / ___ / _____

Hereby I declare that I have fully informed the participant about the current research, encouraged the participant to ask questions, and provided adequate time to answer them. In the case of new information that could influence the permission of the participant, I will inform the participant in a timely fashion. I further ensure that 2 copies of the consent form have been completed: 1 copy to be left with the participant, and 1 copy to be filed with the researcher.

This project has received funding from the European Union's Horizon 2020 research and innovation programme under grant agreement No 779238
For peer review only - http://bmjopen.bmj.com/site/about/guidelines.xhtml

Name of person receiving consent:

Signature:

Job Title: Date: ___ / ___ / ______

2 consent forms to be signed by both parties: 1 copy for the participant; 1 copy for the researcher site file

SAMPLE

For peer review only

This project has received funding from the European Union's Horizon 2020 research and innovation programme under grant agreement No 779238
For peer review only - http://bmjopen.bmj.com/site/about/guidelines.xhtml

Reporting checklist for protocol of a clinical trial.

Based on the SPIRIT guidelines.

Instructions to authors

Complete this checklist by entering the page numbers from your manuscript where readers will find each of the items listed below.

Your article may not currently address all the items on the checklist. Please modify your text to include the missing information. If you are certain that an item does not apply, please write "n/a" and provide a short explanation.

Upload your completed checklist as an extra file when you submit to a journal.

In your methods section, say that you used the SPIRITreporting guidelines, and cite them as:

Chan A-W, Tetzlaff JM, Gøtzsche PC, Altman DG, Mann H, Berlin J, Dickersin K, Hróbjartsson A, Schulz KF, Parulekar WR, Krleža-Jerić K, Laupacis A, Moher D. SPIRIT 2013 Explanation and Elaboration: Guidance for protocols of clinical trials. BMJ. 2013;346:e7586

		Reporting Item	Page Number
**Administrative information**			
Title	#1	Descriptive title identifying the study design, population, interventions, and, if applicable, trial acronym	1
Trial registration	#2a	Trial identifier and registry name. If not yet registered, name of intended registry	2
Trial registration: data set	#2b	All items from the World Health Organization Trial Registration Data Set	n/a
Protocol version	#3	Date and version identifier	n/a
Funding	#4	Sources and types of financial, material, and other support	21
Roles and responsibilities: contributorship	#5a	Names, affiliations, and roles of protocol contributors	21

For peer review only

Roles and responsibilities: sponsor contact information	#5b	Name and contact information for the trial sponsor	22
Roles and responsibilities: sponsor and funder	#5c	Role of study sponsor and funders, if any, in study design; collection, management, analysis, and interpretation of data; writing of the report; and the decision to submit the report for publication, including whether they will have ultimate authority over any of these activities	n/a
Roles and responsibilities: committees	#5d	Composition, roles, and responsibilities of the coordinating centre, steering committee, endpoint adjudication committee, data management team, and other individuals or groups overseeing the trial, if applicable (see Item 21a for data monitoring committee)	6
**Introduction**			
Background and rationale	#6a	Description of research question and justification for undertaking the trial, including summary of relevant studies (published and unpublished) examining benefits and harms for each intervention	4
Background and rationale: choice of comparators	#6b	Explanation for choice of comparators	4
Objectives	#7	Specific objectives or hypotheses	5
Trial design	#8	Description of trial design including type of trial (eg, parallel group, crossover, factorial, single group), allocation ratio, and framework (eg, superiority, equivalence, non-inferiority, exploratory)	6
**Methods: Participants, interventions, and outcomes**			
Study setting	#9	Description of study settings (eg, community clinic, academic hospital) and list of countries where data will be collected. Reference to where list of study sites can be obtained	6,8
Eligibility criteria	#10	Inclusion and exclusion criteria for participants. If applicable, eligibility criteria for study centres and individuals who will	5

For peer review only

		perform the interventions (eg, surgeons, psychotherapists)	
Interventions: description	#11a	Interventions for each group with sufficient detail to allow replication, including how and when they will be administered	6,7
Interventions: modifications	#11b	Criteria for discontinuing or modifying allocated interventions for a given trial participant (eg, drug dose change in response to harms, participant request, or improving / worsening disease)	n/a
Interventions: adherance	#11c	Strategies to improve adherence to intervention protocols, and any procedures for monitoring adherence (eg, drug tablet return; laboratory tests)	6,7
Interventions: concomitant care	#11d	Relevant concomitant care and interventions that are permitted or prohibited during the trial	7
Outcomes	#12	Primary, secondary, and other outcomes, including the specific measurement variable (eg, systolic blood pressure), analysis metric (eg, change from baseline, final value, time to event), method of aggregation (eg, median, proportion), and time point for each outcome. Explanation of the clinical relevance of chosen efficacy and harm outcomes is strongly recommended	7-8
Participant timeline	#13	Time schedule of enrolment, interventions (including any run-ins and washouts), assessments, and visits for participants. A schematic diagram is highly recommended (see Figure)	20
Sample size	#14	Estimated number of participants needed to achieve study objectives and how it was determined, including clinical and statistical assumptions supporting any sample size calculations	10
Recruitment	#15	Strategies for achieving adequate participant enrolment to reach target sample size	5
**Methods: Assignment of interventions (for controlled trials)**			
Allocation: sequence generation	#16a	Method of generating the allocation sequence (eg, computer-generated random numbers), and list of any factors for stratification. To reduce predictability of a random sequence, details of any planned restriction (eg, blocking) should be provided in a separate document that is unavailable to those who enrol participants or assign interventions	9

For peer review only

Allocation concealment mechanism	#16b	Mechanism of implementing the allocation sequence (eg, central telephone; sequentially numbered, opaque, sealed envelopes), describing any steps to conceal the sequence until interventions are assigned	8
Allocation: implementation	#16c	Who will generate the allocation sequence, who will enrol participants, and who will assign participants to interventions	9
Blinding (masking)	#17a	Who will be blinded after assignment to interventions (eg, trial participants, care providers, outcome assessors, data analysts), and how	9
Blinding (masking): emergency unblinding	#17b	If blinded, circumstances under which unblinding is permissible, and procedure for revealing a participant's allocated intervention during the trial	n/a
**Methods: Data collection, management, and analysis**			
Data collection plan	#18a	Plans for assessment and collection of outcome, baseline, and other trial data, including any related processes to promote data quality (eg, duplicate measurements, training of assessors) and a description of study instruments (eg, questionnaires, laboratory tests) along with their reliability and validity, if known. Reference to where data collection forms can be found, if not in the protocol	7-9, 16, 17
Data collection plan: retention	#18b	Plans to promote participant retention and complete follow-up, including list of any outcome data to be collected for participants who discontinue or deviate from intervention protocols	n/a
Data management	#19	Plans for data entry, coding, security, and storage, including any related processes to promote data quality (eg, double data entry; range checks for data values). Reference to where details of data management procedures can be found, if not in the protocol	9
Statistics: outcomes	#20a	Statistical methods for analysing primary and secondary outcomes. Reference to where other details of the statistical analysis plan can be found, if not in the protocol	9,10
Statistics: additional analyses	#20b	Methods for any additional analyses (eg, subgroup and adjusted analyses)	9,10

For peer review only

Statistics: analysis population and missing data	#20c	Definition of analysis population relating to protocol non-adherence (eg, as randomised analysis), and any statistical methods to handle missing data (eg, multiple imputation)	9,10
**Methods: Monitoring**			
Data monitoring: formal committee	#21a	Composition of data monitoring committee (DMC); summary of its role and reporting structure; statement of whether it is independent from the sponsor and competing interests; and reference to where further details about its charter can be found, if not in the protocol. Alternatively, an explanation of why a DMC is not needed	n/a
Data monitoring: interim analysis	#21b	Description of any interim analyses and stopping guidelines, including who will have access to these interim results and make the final decision to terminate the trial	n/a
Harms	#22	Plans for collecting, assessing, reporting, and managing solicited and spontaneously reported adverse events and other unintended effects of trial interventions or trial conduct	9
Auditing	#23	Frequency and procedures for auditing trial conduct, if any, and whether the process will be independent from investigators and the sponsor	n/a
**Ethics and dissemination**			
Research ethics approval	#24	Plans for seeking research ethics committee / institutional review board (REC / IRB) approval	22
Protocol amendments	#25	Plans for communicating important protocol modifications (eg, changes to eligibility criteria, outcomes, analyses) to relevant parties (eg, investigators, REC / IRBs, trial participants, trial registries, journals, regulators)	n/a
Consent or assent	#26a	Who will obtain informed consent or assent from potential trial participants or authorised surrogates, and how (see Item 32)	8
Consent or assent: ancillary studies	#26b	Additional consent provisions for collection and use of participant data and biological specimens in ancillary studies, if applicable	n/a
Confidentiality	#27	How personal information about potential and enrolled participants will be collected, shared, and maintained in order to protect confidentiality before, during, and after the trial	9

For peer review only

Declaration of interests	#28	Financial and other competing interests for principal investigators for the overall trial and each study site	21
Data access	#29	Statement of who will have access to the final trial dataset, and disclosure of contractual agreements that limit such access for investigators	6,9
Ancillary and post trial care	#30	Provisions, if any, for ancillary and post-trial care, and for compensation to those who suffer harm from trial participation	n/a
Dissemination policy: trial results	#31a	Plans for investigators and sponsor to communicate trial results to participants, healthcare professionals, the public, and other relevant groups (eg, via publication, reporting in results databases, or other data sharing arrangements), including any publication restrictions	21
Dissemination policy: authorship	#31b	Authorship eligibility guidelines and any intended use of professional writers	21
Dissemination policy: reproducible research	#31c	Plans, if any, for granting public access to the full protocol, participant-level dataset, and statistical code	n/a
**Appendices**			
Informed consent materials	#32	Model consent form and other related documentation given to participants and authorised surrogates	n/a
Biological specimens	#33	Plans for collection, laboratory evaluation, and storage of biological specimens for genetic or molecular analysis in the current trial and for future use in ancillary studies, if applicable	n/a

Notes:

- 18a: 7-9, 16, 17 The SPIRIT checklist is distributed under the terms of the Creative Commons Attribution License CC-BY-ND 3.0. This checklist was completed on 29. January 2021 using https://www.goodreports.org/, a tool made by the EQUATOR Network in collaboration with Penelope.ai

For peer review only
